# Intrinsic DNA topology as a prioritization metric in genomic fine-mapping studies

**DOI:** 10.1093/nar/gkaa877

**Published:** 2020-10-21

**Authors:** Hannah C Ainsworth, Timothy D Howard, Carl D Langefeld

**Affiliations:** Department of Biostatistics and Data Science, Wake Forest School of Medicine, Winston-Salem, NC 27157, USA; Center for Precision Medicine, Wake Forest School of Medicine, Winston-Salem, NC 27157, USA; Center for Precision Medicine, Wake Forest School of Medicine, Winston-Salem, NC 27157, USA; Department of Biochemistry, Wake Forest School of Medicine, Winston-Salem, NC 27157, USA; Department of Biostatistics and Data Science, Wake Forest School of Medicine, Winston-Salem, NC 27157, USA; Center for Precision Medicine, Wake Forest School of Medicine, Winston-Salem, NC 27157, USA; Comprehensive Cancer Center of Wake Forest Baptist Medical Center, Winston-Salem, NC 27157, USA

## Abstract

In genomic fine-mapping studies, some approaches leverage annotation data to prioritize likely functional polymorphisms. However, existing annotation resources can present challenges as many lack information for novel variants and/or may be uninformative for non-coding regions. We propose a novel annotation source, sequence-dependent DNA topology, as a prioritization metric for fine-mapping. DNA topology and function are well-intertwined, and as an intrinsic DNA property, it is readily applicable to any genomic region. Here, we constructed and applied Minor Groove Width (MGW) as a prioritization metric. Using an established MGW-prediction method, we generated a MGW census for 199 038 197 SNPs across the human genome. Summarizing a SNP’s change in MGW (ΔMGW) as a Euclidean distance, ΔMGW exhibited a strongly right-skewed distribution, highlighting the infrequency of SNPs that generate dissimilar shape profiles. We hypothesized that phenotypically-associated SNPs can be prioritized by ΔMGW. We tested this hypothesis in 116 regions analyzed by a Massively Parallel Reporter Assay and observed enrichment of large ΔMGW for functional polymorphisms (*P* = 0.0007). To illustrate application in fine-mapping studies, we applied our MGW-prioritization approach to three non-coding regions associated with systemic lupus erythematosus. Together, this study presents the first usage of sequence-dependent DNA topology as a prioritization metric in genomic association studies.

## INTRODUCTION

Genetic association studies have successfully identified thousands of loci associated with a broad range of phenotypes (1). However, despite the abundance of these genomic associations, analytic challenges have largely hindered identification of the specific genomic drivers of disease (2–4). First, linkage disequilibrium (LD) constitutes a major analytic challenge, as highly correlated variants exhibit comparable evidence of association, making it difficult to statistically isolate causal polymorphisms. Second, many associated single nucleotide polymorphisms (SNPs) reside in non-coding regions, occluding functional relevance without additional context and information. Even with increased sample sizes and variant coverage, these challenges remain (2–5). In-depth functional analyses are not practical for a large number of variants, and thus, there remains the need to effectively prioritize the most likely causal variants for follow-up studies and approaches (e.g. CRISPR).

To prioritize potential causal variants, association results can be weighted by functional information (e.g. histone modifications, CHIP-seq) from publicly available resources (5–8). This approach has been successful in reducing and refining associated variants, and there are a growing number of tools and methods that integrate functional data with genomic association studies (6,9–13). Importantly, in these methods, the choice of annotation and potential database bias are strong factors for consideration as missing or incomplete functional data could result in down-weighting causal polymorphisms that are absent from the resource. These challenges particularly arise for regions and variants that are previously unstudied and/or have unknown functional implications. Additionally, many publicly available annotation resources are based primarily on European data and may offer limited information for genetic studies in non-European ancestries (e.g. novel regions) (14,15). Such limitations can reduce the rate of progress in understanding the functional impact of ancestry-specific associations and perpetuate health disparities (16,17). To alleviate some of these biases imposed by external datasets, we propose a prioritization approach that leverages information intrinsic to the DNA itself, sequence-dependent DNA topology.

**Figure 1.**
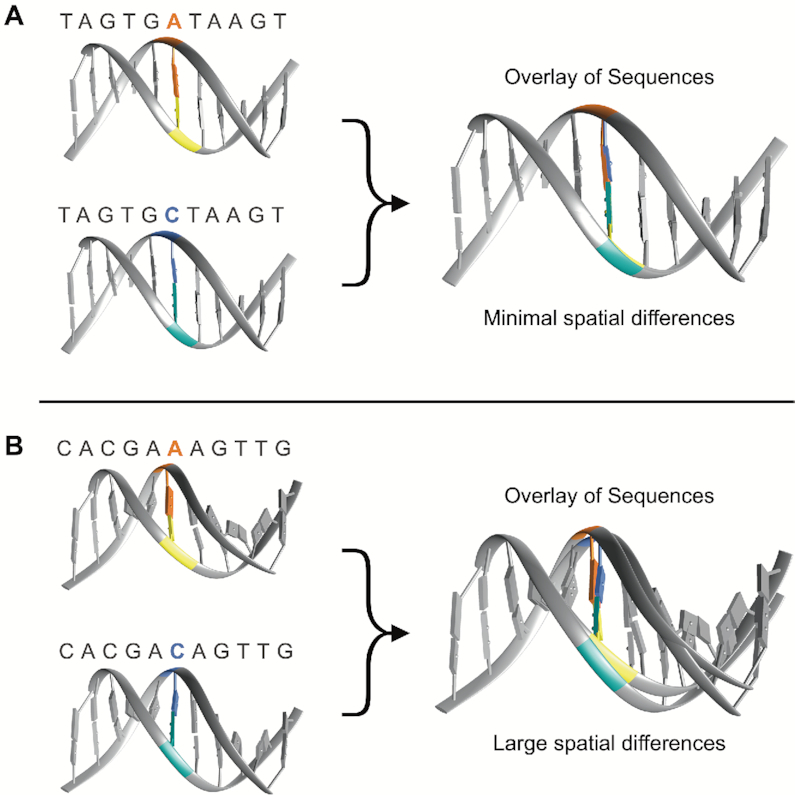
Single nucleotide substitutions in a sequence can impose large or small changes on local DNA shape, dependent on the flanking sequence. (**A**) A single A/C substitution within a sequence generates minimal spatial differences. (**B**) A single A/C substitution within a sequence imposes large spatial differences.

From chromatin conformation to selective protein binding (18–26), DNA is a highly dynamic macromolecule with structure inherently linked to function. Sequence-dependent DNA topology (or shape) refers to the geometric parameters (measured in Angstroms or degrees) between successive nucleotides in a DNA sequence (24,27–29). The sequence dependency of these spatial measures (Figure 1) has been well-studied and in recent years, increasingly connected to various functional implications including protein binding, DNA stability, and methylation (18,20,21,23,30–38). High-throughput DNA shape prediction methods now enable exploration of DNA topology on a genome-wide scale, and thus, provide new opportunities in association studies (24,39).

**Figure 2.**
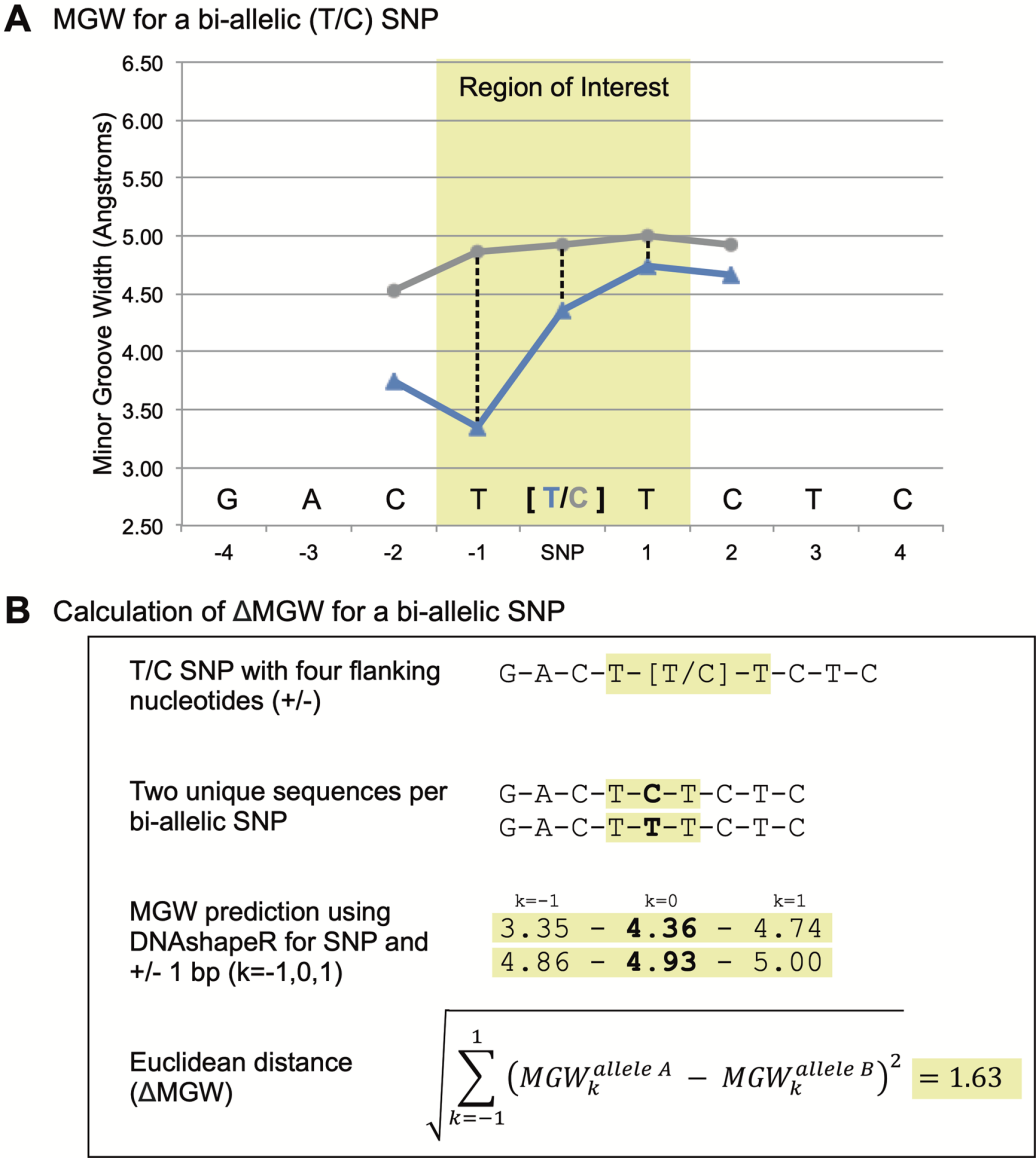
Generation of ΔMGW for a SNP. (**A**) Minor groove width measures are plotted for the two sequences generated by a specific bi-allelic T/C SNP. For a given SNP, the flanking sequence (±4 bp) was used as input for DNAshapeR (via Bioconductor) which calculates MGW along a rolling sequence window. For a 9-mer sequence, the MGW can be consistently provided at the SNP’s position ± one nucleotide which is highlighted in yellow and labeled as the ‘region of interest’. Expanding this region to additional nucleotides would require a longer input sequence and increases chance of additional genetic variants within the input (and introducing additional variability). Although the two sequences for a SNP only differ at one nucleotide (at the SNP position), the impact on MGW carries through adjacent bases. Thus, ΔMGW was calculated to capture the change in MGW for a SNP by incorporating information at the SNP’s position and ±1 base pair (dashed lines). (**B**) Workflow for calculating the ΔMGW for a bi-allelic SNP. This method captures the change in MGW at the SNP position and ±1 base pair. This Euclidean distance captures ΔMGW as a measure of magnitude (in Ångstroms).

This study presents using sequence-dependent DNA topology as a prioritization metric in genomic association studies. Here, we focused on minor groove width (MGW), which measures the distance (angstroms, Å) between the sugar phosphate backbone of the forward and reverse strands. For each SNP, we analyzed its change in minor groove width (ΔMGW) to evaluate whether the SNP’s alleles created similar or divergent MGW profiles. MGW has been implicated in numerous protein binding studies and used in transcription factor binding prediction algorithms (18,20,24,32,34,36,37,40,41). Recently it was studied in the context of purifying selection, where ‘shape disrupting variants’ (examples in Figures 1 and 2) tend to be less common in functional regions (shape-preserving polymorphisms being more frequent) (42). Thus, we proposed that if a phenotypically-associated SNP also yields a large ΔMGW, it is more likely to be causal as a function of divergent shape profiles.

We specifically hypothesized that highly correlated SNPs in a phenotype-associated region can be functionally prioritized using each SNP’s magnitude of ΔMGW. We evaluated this hypothesis in four stages. First, using an established MGW-prediction algorithm (39), we generated the complete sample space for ΔMGW for all possible input sequences. Second, we evaluated the observed frequency of ΔMGW across the human genome using bi-allelic SNPs in the dbSNP SNP150 dataset. Third, while large deviations in ΔMGW could impact several functional mechanisms, we explored patterns between ΔMGW and allele-specific activity through a previously published Massively Parallel Reporter Assay (MPRA) (43). Finally, to illustrate our method, we applied ΔMGW-prioritization (through both frequentist and Bayesian approaches) in three genomic regions previously associated with systemic lupus erythematosus (SLE) (44). For each region, we aimed to reduce the number of potentially functional variants amidst the previously identified LD block of association (44). Together, this manuscript provides a detailed description, summary, and application of an intrinsic DNA property to enhance fine-mapping studies.

## MATERIALS AND METHODS

### Calculation of ΔMGW for a bi-allelic SNP

The predicted MGW for a given sequence was obtained using the DNAshapeR package (https://bioconductor.org/packages/release/bioc/html/DNAshapeR.html), available through Bioconductor (39). DNAshapeR calculates DNA features using Monte Carlo simulations for nucleotide structure based on DNA sequence fragments. DNA feature predictions are based on a rolling window of five nucleotides for a given n-length sequence. For this study, to capture the MGW at a SNP, we used the four flanking (up and downstream) nucleotides (9-mer sequence) as input. Each bi-allelic SNP produces two unique 9-mer sequences (one sequence for each allele) and thus, both of a SNP’s sequences were submitted to DNAshapeR to obtain the corresponding feature vectors for MGW. The MGW was retained for the nucleotide at the SNP’s position as well as ±1 nucleotides. Capturing MGW for additional bases would require longer input sequences, which could introduce additional variability (e.g. SNPs within the flanking sequence). The ΔMGW was calculated as a Euclidean distance for the SNP and ±1 nucleotides (Figure 2).

### Generation of ΔMGW sample space

**Figure 3.**
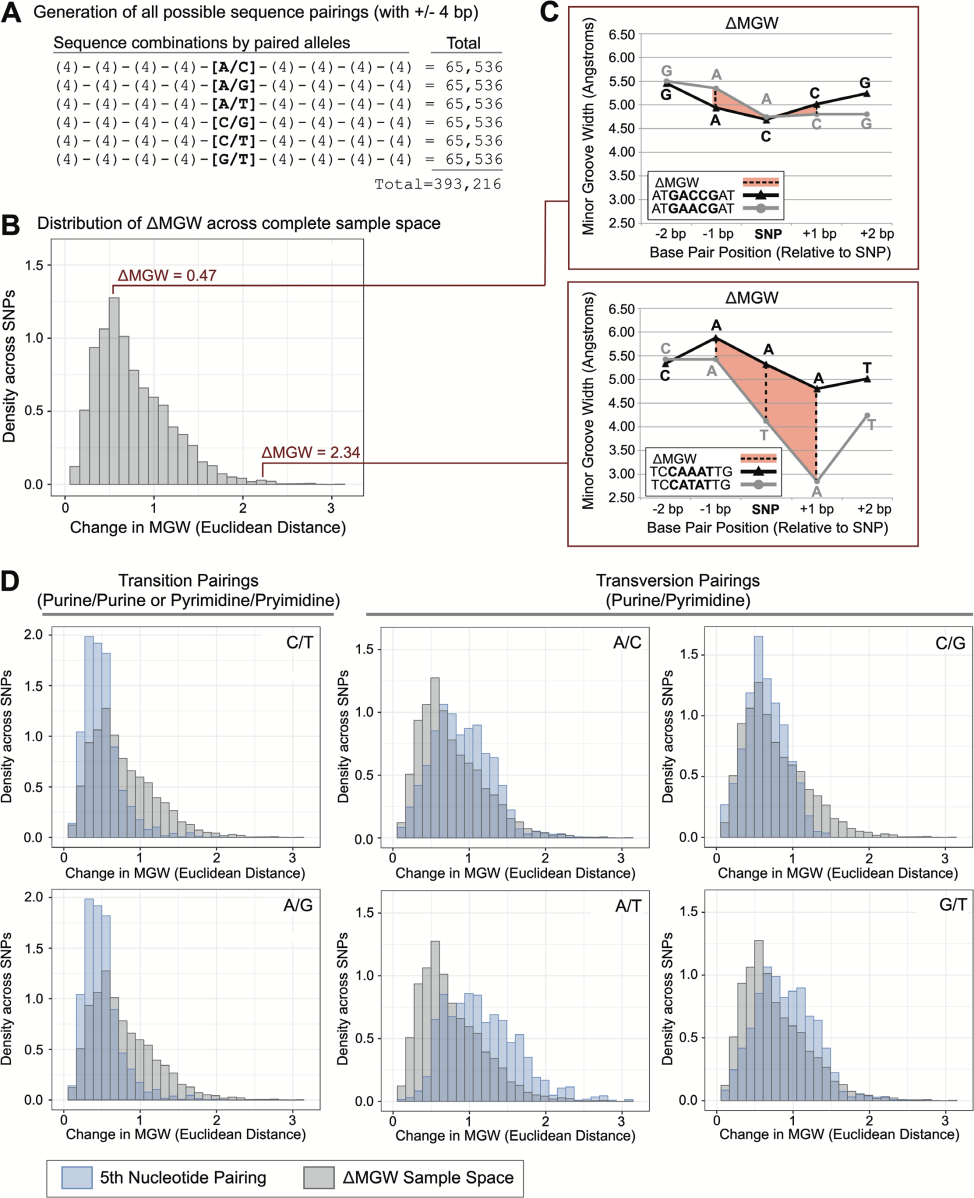
Summarization of ΔMGW across the complete sample space (**A**) ΔMGW sample space was constructed on six allele pairings (A/C, A/G, A/T, C/G, C/T, G/T) with all possible combinations for flanking ±4 bp. This yielded 393 216 paired sequences that were evaluated for ΔMGW. (**B**) The distribution of ΔMGW for the 393 216 paired sequences, these summary statistics are listed in Table 1. (**C**) Two randomly selected paired sequences from the average and right tail of the ΔMGW distribution are shown. Sequences are plotted with their respective MGW values (Å). ΔMGW is calculated as a Euclidean distance, which captures the change in MGW (dashed lines) at the SNP position and ±1 bp (highlighted in orange). ATGA[C/A]CGAT exhibits a small ΔMGW, at 0.47 Å while TCCA[T/A]ATTG yields a large change in MGW (2.34 Å) which we would hypothesize to have greater potential for functional consequence if also associated with a phenotype (two-parameter hypothesis). (**D**) The ΔMGW distribution for all paired sequences (gray) is shown superimposed on the ΔMGW distributions by 5th nucleotide alleles (blue). Transition pairings (C/T, A/G) have a more strongly skewed distribution with a smaller average ΔMGW compared to transversion pairings (A/C, A/T, C/G, G/T) (Table 1). Pairings that represent complimentary sequences (C/T – A/G and A/C – T/G) exhibit the same distributions of ΔMGW, as expected.

To calculate the entire sample space for ΔMGW, we generated a dataset of all possible 9-mer sequences. All possible combinations of adenine, cytosine, guanine and thymine, generated 4^9^ (262 144) 9-mer sequences. From this dataset, all possible bi-allelic pairings (A/C, A/G, A/T, C/G, C/T, G/T) were created on the fifth nucleotide of each sequence (‘SNP position’) while holding the flanking nucleotides constant, generating 393 216 9-mer pairings. These 9-mer pairings represent every possible sequence combination that could be observed for a bi-allelic SNP (Figure 3). These paired sequences were evaluated for ΔMGW using the previously described method.

### Visualization of DNA sequences

DNA shape measures, provided by DNAshapeR, were submitted as a parameter file to the 3D-Dart webportal (http://milou.science.uu.nl/services/3DDART/) for a ‘BDNA nucleic acid’ (45). Resulting pdb files from 3D-Dart were then visualized using Chimera (https://www.cgl.ucsf.edu/chimera/) (46).

### Curating dbSNPs150 database

The NCBI hg19 dbSNPs150 data file (snp150.txt.gz) was downloaded via UCSC GoldenPath (hgdownload.cse.ucsc.edu) on July 6, 2018 (47). Insertion-deletions, tri-allelic, quad-allelic, and multiple nucleotide polymorphisms were excluded. Retained bi-allelic SNPs were limited to those located on chromosomes 1–22 and X. Any SNPs that were labeled with ‘Unusual Conditions’ as defined by UCSC were excluded, as these indicate possible discrepancies among alleles and/or potential mapping issues (e.g. SNP flanking sequence aligns to more than one location in the reference assembly) (47,48). The pruned bi-allelic dataset contained 199,038,272 SNPs.

For dbSNP 150 data, each SNP’s flanking sequence of four nucleotides was retrieved from the Human Reference Genome (downloaded from the Build 37 GATK resource bundle on October 2017; available by ftp.broadinstitute.org/bundle) (49) using SAMTOOLS. For each SNP, the dbSNP ‘Strand’ variable was used to inform if the alleles reported by dbSNP aligned with the reference genome. All SNPs were successfully queried against the reference genome. There were 75 SNPs that contained at least one flanking base encoded as ‘N’ (any base) and were excluded from summarizations, leaving a final dataset of 199 038 197 SNPs. The ΔMGW for these sequences were obtained as described above.

### Curating data from a previously published MPRA study

MPRA data was downloaded from Supplementary Table S1 of a previously published study by Tewhey *et al.* (43). The table of 39 478 variants tested by the MPRA was filtered to 4335 ‘active sequences’ (e.g. those that showed detectable expression). Duplicate variants (*n* = 740) were removed to retain the one with the most significant allelic skewing. Insertion-deletions were also excluded. Any remaining variants that did not meet the filtering criteria for ΔMGW analyses of the dbSNP150 data (as previously described) were pruned, leaving bi-allelic SNPs without ‘unusual mapping conditions’ by the UCSC Table browser. The final dataset of MPRA data contained 2819 SNPs.

Genomic regions were defined by the SNP with the most significant MPRA allelic-skewing *P*-value and all (filtered) SNPs tested within 500kb upstream and downstream of the top variant. To test for global trends of enrichment, genomic regions were required to have at least 5 SNPs (*n* = 136 regions) and were retained only if there was at least one SNP in the region that met FDR significance for allelic skewing as defined by Tewhey *et al.* (116 regions; 1368 SNPs). For the final dataset, ΔMGW values were obtained based on rsID lookups in the SNP 150 data, as previously described.

### SLE Immunochip Data for fine-mapping analyses

Genomic data for fine-mapping analyses came from the published trans-ancestral SLE Immunochip study; genotype calling and genomic quality control methods were previously described (44). This data includes three ancestries, European Ancestry (EA), African Ancestry (AA) and Hispanic Ancestry (HA), with large case-control counts: EA (6748; 11 516), AA (2970; 2452) and HA (1872; 2016).

Genomic regions were named for the genes in physical proximity to the region of association. Non-HLA genomic regions were selected for fine-mapping if the region contained SNPs reaching genome-wide significance (*P* < 5 × 10^−8^) in at least two ancestry-specific analyses (44). We also limited our analyses to regions where the top associations mapped to non-coding regions (e.g. introns, intergenic), where we hypothesize DNA topology might provide novel insight to the fine-mapping analyses. Genomic regions containing *FAM167A-BLK* (8p23), *STAT4* (2q32) and *TNIP1* (5q33) met these *a priori* criteria. Quality controlled genomic data for these regions were extracted using a 250 kb window around the previously reported top association from the Immunochip analysis (44).

SNPs from the selected genomic regions were queried against the human reference genome (as previously described for the dbSNP 150 database) to retrieve the four flanking nucleotides. Each SNP’s strand information (based on Illumina Infinium Immunochip manifest file) was utilized to ensure that the corresponding alleles appropriately aligned with the reference genome. Using the 9-mers created by the SNP’s alleles and flanking nucleotides, the ΔMGW was calculated using DNAshapeR, as previously described (39).

### Statistical analyses

#### Single-SNP associations

Single-SNP associations were previously reported and described in the transancestral SLE Immunochip study (44).

#### SKAT analyses

The previous single-SNP logistic regression analyses (44) did not incorporate SNP-specific weights/information. Thus, SNPs in high LD yielded comparable association values. The Sequence Kernel Association Test (SKAT) is a regression approach that was designed to handle covariates and SNP-specific weights through a weighted linear kernel (50). It was shown that well-selected SNP weights can yield better statistical power (e.g. increasing weight of functional variants) (50). SKAT was originally developed to leverage minor allele frequency (MAF), as the weighting scheme in rare variant studies; however, the SKAT framework is a general method that can accommodate any user-specified SNP weights (50). Here, we used ΔMGW as the weighting scheme. A variation of SKAT is the Optimal unified test which combines both SKAT and the burden test (SKAT-O) (12). The SKAT-O test statistic is a weighted average of the SKAT and burden test statistics and can be beneficial when applying to genomic regions where one test may be better powered than another (51). Primary advantages of burden tests occur when a large number of variants are causal and for smaller sample sizes (SKAT loses power in small sample sizes, <2000 cases and controls). Generally, burden tests do not perform as well as SKAT when a large proportion of the variants are non-causal (12,50,51). In this study, our datasets are large (AA: 5422; EA: 18 264; HA: 2016), and we expect many of the highly associated SNPs in LD to be non-causal; thus, in this scenario we selected SKAT to be more appropriate, which is consistent with published power calculations and simulations (12,50,51). SKAT was applied to genomic regions through its implementation in the R package, SKAT (https://CRAN.R-project.org/package=SKAT). For each genomic region, the model parameters and residuals were calculated for SKAT using SKAT_Null_Model() for a dichotomous outcome (case/controls status) and previously described (44) population-specific factors (to account for admixture). Since all datasets (AA, EA, and HA) had a sample size >2000 cases and controls, no small-sample adjustment was applied. Within each genomic region, adjacent 5-SNP windows were generated, offset by 1 SNP. Each window was evaluated using the SKATbinary() with method = SKAT and a linear-weighted kernel with SNPs weighted by their ΔMGW. To evaluate consistency of the results (e.g. for SNPs outside of the main peak of association), genomic regions were also evaluated using equal-weighting for all SNPs. Given the small window size (*n* = 5 SNPs), we expect a large proportion of each window to contain non-causal SNPs, further supporting our selection of SKAT. For comparison, we also applied SKAT-O but noted minimal differences on the final outcome. To localize the top association signals to each SNP, SNP-window *P*-values were treated as a SNP prioritization metric by generating the geometric mean of −log_10_(*P*-values) across windows containing each SNP. That is, the prioritization metric was calculated using the *P*-value for each SKAT analysis window (p_i_) that contained the *k*th SNP (*n* analysis windows). With the exception of the first and last five SNPs in a region, each SNP_*k*_ was included in five analysis windows (n = 5). Thus, for each SNP *k*, we calculated its prioritization metric as:

(1) $$\begin{equation*}{\rm Prioritization}\ {\rm Metric}\ {{\rm SNP}_{k }} = - {\log_{10}}{\left( {\mathop \prod \limits_{i = 1}^n {p_i}} \right)^{\frac{1}{n}}}\end{equation*}$$

#### Bayesian approach: credible SNP sets

Frequentist approaches, such as those implemented SKAT or single-SNP logistic regression analyses are widely utilized; however, their resulting *P*-values are not without limitations (52). For one, *P*-values do not capture the confidence of a particular association. Furthermore, they are more dependent on factors such as the power of the statistical test (influenced by sample size and other variables). Bayesian methods offer an alternative approach; here, Bayes factors are used, capturing the ratio of probabilities between the null and alternative hypotheses.

As a comparison to the frequentist approaches, we used SNPTEST to generate the Bayes factors (BF), using the score test and additive genotype modeling (53). Posterior probabilities for a given SNP *k*, were then calculated using method published by the Welcome Trust Case Control Consortium (54). For SNPs 1-j in the region, the posterior probability for each SNP *k*, was calculated by:

(2) $$\begin{equation*}{\rm Posterior}\ {\rm Probability}\ {\rm for}\ {{\rm SNP}_k} = \frac{{{{\rm BF}_k}}}{{\mathop \sum \nolimits_j {{\rm BF}_j}}}\end{equation*}$$

Using these posterior probabilities, the 95% credible set was determined for each region. This test assumes only one causal SNP in the region and places equal *a priori* probabilities that the causal SNP is any one of the analyzed SNPs (54). In this study, we applied this method to previously defined regions (44) where we hypothesized the association signal is driven by one SNP.

Like the single-SNP logistic regression analyses, this Bayesian analysis is not weighted by functional data. Thus, for a ΔMGW-weighted analysis, a derived credible set was generated from posterior probabilities that accounted for each SNP’s ΔMGW through *ad hoc* weighting, where the posterior probability for a given SNP *k*, was calculated by weighting the Bayes factor by ΔMGW_*k*_ divided by the weighted average of Bayes factors for SNPs 1−*j* in the region. Here, the derived posterior probability for each SNP *k*, is:

(3) $$\begin{eqnarray*} && {\rm Derived}\ {\rm Posterior}\ {\rm Probability}\ {\rm for} \ {{\rm SNP}_k} \nonumber \\ && = \frac{{{{\rm BF}_k}\ {\rm{\Delta MG}}{{\rm{W}}_k}}}{{\mathop \sum \nolimits_j {{\rm BF}_j}\ {\rm{\Delta MG}}{{\rm{W}}_j}}} \end{eqnarray*}$$

Using these values, the derived 95% credible SNP sets were generated and compared with the unweighted 95% credible SNP sets. This methodology enabled weighting by a continuous variable versus existing methods designed for dichotomous (presence/absence of functional annotation) SNP weights (55).

#### Correlation between ΔMGW and Log_2_ fold change in MPRA data

To evaluate the relationship between ΔMGW and allele-specific activity, Pearson correlation coefficient (*r*) between ΔMGW and the absolute value of log_2_ fold change by MPRA (‘LogSkew_Comb’ from downloaded data) was computed for each of the 116 genomic regions (43). We hypothesize larger changes of ΔMGW to be positively correlated with larger magnitudes of allele-dependent functional activity (*r* > 0). To test for enrichment of *r* > 0, we compared positive and negative *r* counts at iterative thresholds of |*r*| in increments of 0.1 magnitude using the two-sided binomial test. To illustrate the resulting mixture distribution (a null distribution with an enrichment of a subset under the alternative), we fit a normal curve with mean of zero and a variance estimated by the 6σ method (i.e. range divided by 6) applied to the negative correlations (56). Under the null hypothesis, *r* will be symmetric about zero and the standard deviation can be estimated using the range from zero to the smallest negative value and dividing this value by three.

#### ΔMGW ranking of top allelic skewing SNPs

For each of the 116 genomic regions, SNPs were ranked from smallest to largest according to ΔMGW and these ranks were converted to percentiles. These ΔMGW ranks were summarized across each region's top MPRA SNP (SNP yielding the largest log_2_ fold change in each region) (*n* = 116 SNPs). We computed a chi-squared goodness-of-fit test under the null hypothesis of no relationship between ΔMGW and log_2_ fold change.

### Functional annotation

To evaluate the functional plausibility for an identified variant, several publicly available resources were referenced. For variant associations with gene expression (eQTL status), the Genotype-Tissue Expression (GTEx) dataset, version 8 was queried at gtexportal.org (57). SNPs were also queried using the SCREEN (Search Candidate *cis*-Regulatory Elements by Encode, http://screen.encodeproject.org) (58,59). Built using Encode data, SCREEN (b38) evaluates if a given genomic coordinate (e.g. based on rsID) resides in a Candidate *cis*-Regulatory Element (ccRE). ccREs are designated based on evidence from DNase hypersensitivity sites, H3K4me3 and H3K27ac histone activity, and CTCF-binding data. SCREEN contains 1.31 million ccREs, correlating to 20.8% of the mappable human genome (http://screen.encodeproject.org). For both, GTEx and SCREEN, functional searches were agnostic to tissue type. Genomic variants were also evaluated for evidence of long-range DNA interaction via Hi-C data (hg19) available through the Yue Lab 3D Genome Browser (http://promoter.bx.psu.edu/hi-c/) (60). Similar to the ccRE search, SNPs were queried to see if they resided in a genome region that exhibited long-range chromatin interactions. The Yue Lab's Capture Hi-C data offers information across 19 cell line options. We evaluated immune-related cell types: naïve B-Cells, CD4_Total (CD4 activated and Naïve), CD8 naïve, monocytes, and neutrophils.

## RESULTS

### For ΔMGW, SNPs in the human genome exhibit a stronger right skewed distribution in comparison to the complete sample space

In the complete sample space of ΔMGW, ΔMGW values ranged from 0.00 to 3.16 Å, with a mean of 0.77 Å and a median of 0.68 Å (Table 1). The overall data exhibited a right-skewed distribution (Figure 3). Transition pairings (purine/purine or pyrimidine/pyrimidine) yielded the smallest changes in ΔMGW, while transversion pairings (purine/pyrimidine) produced the largest. Complimentary allele pairs (i.e. A/G & T/C; A/C & T/G) yielded the same ΔMGW values (Table 1). A/T allele pairings presented the largest ΔMGW with a mean of 1.16 Å (SD, 0.47) (Figure 3).

**Table 1.** Summary statistics for the complete ΔMGW (Å) sample space

				Percentiles (ΔMGW)				
5th Nucleotide pairing^a^	Min.	Max.	Mean ± SD^b^	10th	25th	(Median) 50th	75th	90th
A/C	0.03	2.74	0.90 ± 0.39	0.42	0.62	0.86	1.16	1.51
A/G	0.05	2.07	0.50 ± 0.25	0.25	0.34	0.46	0.60	0.95
A/T	0.07	3.16	1.16 ± 0.48	0.60	0.80	1.11	1.46	1.99
C/G	0.00	1.44	0.64 ± 0.27	0.29	0.46	0.62	0.83	1.10
C/T	0.05	2.07	0.50 ± 0.25	0.25	0.34	0.46	0.60	0.95
G/T	0.03	2.74	0.90 ± 0.39	0.42	0.62	0.86	1.15	1.51
Complete sample space	0.00	3.16	0.77 ± 0.42	0.31	0.46	0.67	1.01	1.55

^a^Pairings generated by 5th nucleotide in 9-mer sequence, all other nucleotides held constant. Each allelic pairing contains 65 536 paired sequences, summing to 393 216 pairings for the complete sample space (as shown in Figure 3).

^b^Standard deviation.

**Figure 4.**
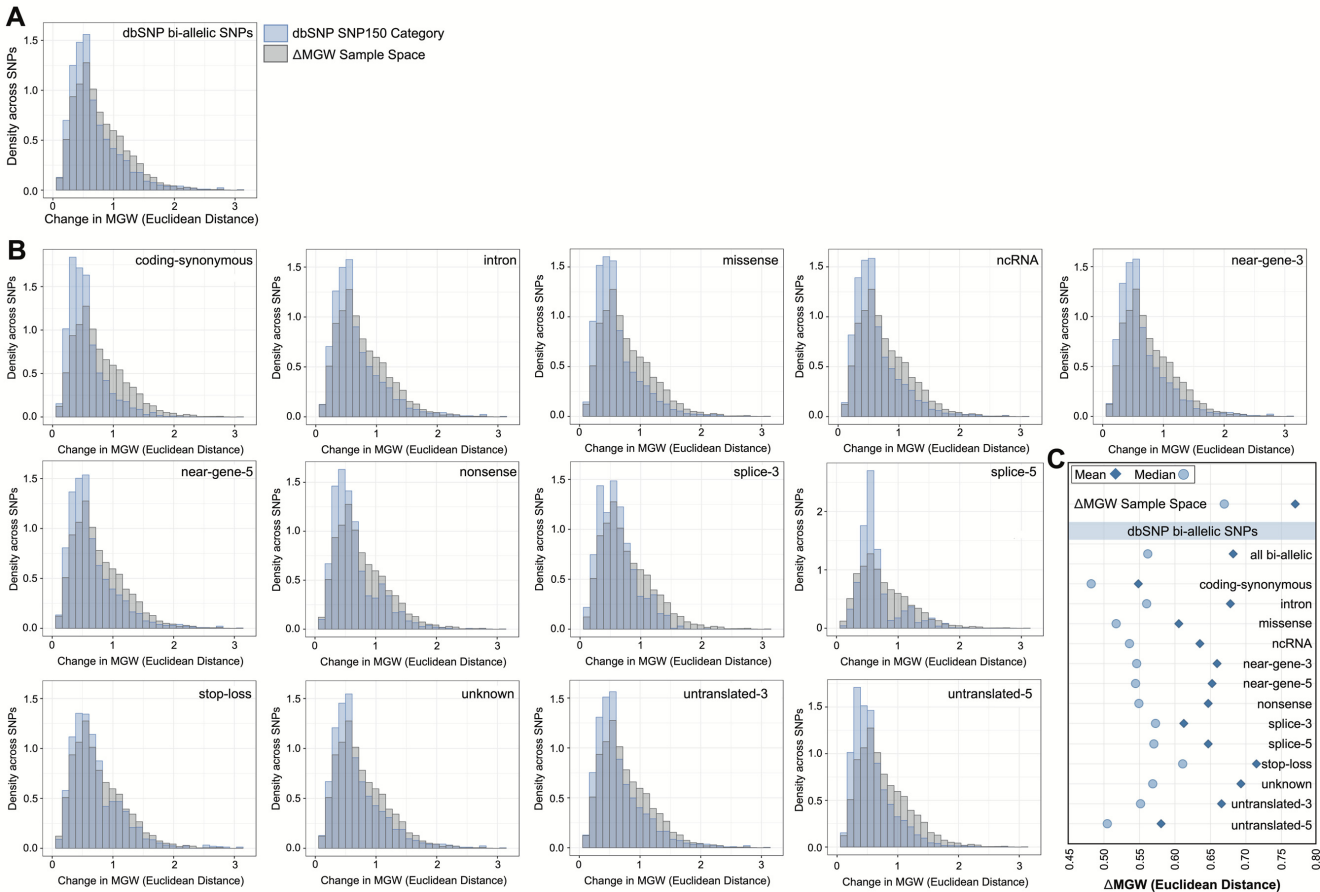
Summarization of ΔMGW across the human genome using bi-allelic SNPs from dbSNP SNP150. (**A**) Comparison of ΔMGW sample space (Figure 3) and the observed ΔMGW from SNPs across the genome (via dbSNP). Distribution of ΔMGW is shown in blue for observed bi-allelic SNPs from the SNP150 dataset (*n* = 199 038 197 SNPs). The ΔMGW sample space distribution from Figure 3 is plotted in gray (*n* = 393 216 paired sequences). The observed ΔMGW across genomic SNPs showed a stronger right skewed distribution than what would be expected from a random sampling of the entire sample space of all-possible sequences. Only small numbers of SNPs elicit large magnitudes of ΔMGW. (**B**) ΔMGW distributions are similarly shown for SNP subsets, by NCBI function (exclusive NCBI function label for each SNP, see Materials and Methods). Again, each distribution is superimposed with the distribution from the ΔMGW sample space (shown in gray). Some NCBI defined SNP functions have specific sequence requirements (Supplementary Table S2) and these are reflected in the ΔMGW distributions which are also sequence-dependent (e.g. splice-6, nonsense). (**C**) The mean and median ΔMGW for each SNP category. All dbSNP SNP categories have significantly lower mean and median compared to the ΔMGW sample space (Tables 1-2). Coding-synonymous SNPs have the smallest magnitudes of ΔMGW, compared to all other categories.

We compared the ΔMGW sample space statistics to the observed frequencies of ΔMGW across the human genome using dbSNP data. The hg19 download of NCBI dbSNP150 contained 234 104 110 entries. After pruning to high quality, bi-allelic SNPs, 199 038 197 polymorphisms remained. For these SNPs, there was a mean ΔMGW of 0.68 Å with a standard deviation of 0.43. In comparison to the ΔMGW sample space, SNPs across the genome exhibited a stronger, right-skewed distribution of ΔMGW (Figure 4). Transition SNPs are more likely to occur (61,62), and this is consistent with our SNP150 summarizations, where transition SNPs comprised 66.43% of the dataset (Supplementary Table S1). Our ΔMGW sample space summarization showed that transition allele pairings had the smallest change in ΔMGW (Table 1); thus, the decreased average in ΔMGW dbSNP data is as hypothesized and illustrates the high prevalence of shape-preserving SNPs in the genome. To evaluate patterns in ΔMGW by SNP function (i.e. missense, intron, coding-synonymous), SNPs with a single NCBI-designation (see Methods and Materials) were subset and summarized (Table 2, Figure 4). Notably, some SNP categories are limited to specific sequence combinations (63) (i.e. stop-loss, Supplementary Table S2), which were reflected in the SNP-function-specific patterns of ΔMGW (Figure 4). Coding-synonymous SNPs exhibited the smallest overall change in ΔMGW (mean = 0.48 Å). SNPs defined by NCBI as ‘unknown’ and intronic are not constrained to specific sequences (by definition) and comprised the two largest categories (*n*_unknown_ = 99 004 130; *n*_intron_ = 84 909 115) and yielded ΔMGW means of 0.69 and 0.56 Å, respectively.

**Table 2.** Summary statistics for ΔMGW (Å) across bi-allelic SNPs in dbSNP SNP150 dataset

					Percentiles				
SNP category^a^	*N*	Min.	Max.	Mean ± SD^b^	10th	25th	(Median) 50th	75th	90th
dbSNP SNP150^c^	199 038 197	0.00	3.16	0.68 ± 0.43	0.28	0.40	0.56	0.86	1.22
coding-synonymous	1 178 980	0.00	2.58	0.55 ± 0.30	0.25	0.34	0.48	0.65	0.95
intron	84 909 115	0.00	3.16	0.68 ±0.42	0.28	0.40	0.56	0.85	1.21
missense	2 345 831	0.00	3.16	0.61 ± 0.36	0.26	0.36	0.52	0.74	1.11
ncRNA	499 593	0.00	3.16	0.63 ± 0.38	0.27	0.38	0.54	0.79	1.15
near-gene-3	654 589	0.00	3.16	0.66 ±0.41	0.28	0.39	0.55	0.81	1.18
near-gene-5	2 487 192	0.00	3.16	0.65 ± 0.41	0.27	0.38	0.54	0.81	1.17
nonsense	66 275	0.00	3.16	0.65 ± 0.37	0.28	0.39	0.55	0.79	1.18
splice-3	25 401	0.01	2.07	0.61 ± 0.31	0.28	0.37	0.57	0.77	1.08
splice-5	28 983	0.00	2.74	0.65 ± 0.31	0.37	0.46	0.57	0.71	1.15
stop-loss	2225	0.03	3.16	0.71 ± 0.42	0.31	0.42	0.61	0.91	1.46
unknown	99 004 130	0.00	3.16	0.69 ± 0.43	0.29	0.41	0.57	0.88	1.24
untranslated-3	1 299 685	0.00	3.16	0.67 ± 0.41	0.28	0.39	0.55	0.83	1.19
untranslated-5	181 208	0.00	3.16	0.58 ± 0.33	0.25	0.34	0.50	0.72	1.05

^a^NCBI-function specific categories represent exclusive categories of SNPs.

^b^Standard deviation.

^c^dbSNP 150 (hg19) bi-allelic SNPs, excluding insertion-deletions, MNPs, and SNPs labeled with unusual mapping conditions by the UCSC Table browser.

### Regional correlation identified between ΔMGW and reporter gene expression in MPRA data

While changes in MGW have been implicated in a number of targeted functional studies, we aimed to identify patterns between ΔMGW and function on a global scale. The Massively Parallel Reporter Assay (MPRA) for finding expression-modulating variants presents an ideal framework for evaluating patterns between intrinsic ΔMGW and functionality. Allelic skewing is identified by comparing reporter assay activity between two oligonucleotides which are identical except at the selected SNP locus. As such, MPRA can be used to experimentally prioritize variants that are in high LD. We utilized a published MPRA dataset (43) to test for associations between ΔMGW and allelic skewing, hypothesizing that evidence of allelic skewing would correlate with larger magnitudes of ΔMGW when prioritizing SNPs in a genomic region.

**Figure 5.**
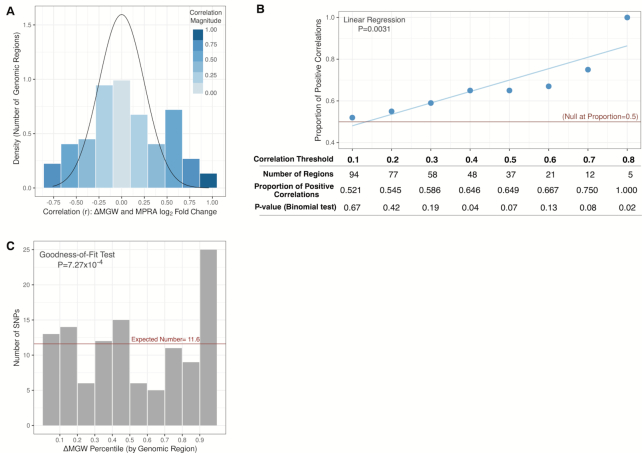
Correlation between ΔMGW and allele-specific activity as measured by log_2_ fold change in a Massively Parallel Reporter Assay. (**A**) Correlations between ΔMGW and log_2_ fold change for SNPs (*n* = 116 genomic regions. Distribution of correlation estimates (*r*) shows enrichment for genomic regions exhibiting large positive correlations between magnitude of allele-specific activity and magnitude of ΔMGW. (**B**) The proportion of positive correlations between log_2_ fold change and ΔMGW shows enrichment at increasing thresholds of correlation estimates. Under null the null hypothesis we would expect to see equivalent (0.5) proportion of negative and positive correlation estimates at any threshold of *r*, shown as a red line. Here, we see enrichment of positive correlations. Globally, there is a significant (*P* = 0.0031) trend with increasing *r* thresholds, up to r = 0.8 where all five regions show positive correlation between ΔMGW and allele-specific activity. (**C**) Regional ΔMGW percentile for SNPs showing the greatest allele-specific activity in 116 genomic regions. For each region, we summarized the relative ΔMGW for the SNP with the largest magnitude of log_2_ fold change. Under the null (no relationship between ΔMGW and allele-specific activity), we would expect a uniform distribution of ΔMGW with an expected value of 11.6 per bin (116/10). Instead, we observe significant departure from the uniform distribution based on a chi-squared goodness-of-fit test (*P* = 0.0007). There was strong enrichment for SNPs (with the greatest allele-specific activity) to also exhibit the largest magnitude of ΔMGW in the region.

MPRA data from 1,368 SNPs spanning 116 genomic regions were evaluated for patterns between ΔMGW and reporter assay activity as measured by the absolute value of log_2_ fold change (Supplementary Table S3; Figure S1). The distribution of the Pearson correlation coefficient (*r*) shows a mixture distribution with a subset of the regions not showing an enrichment of larger, positive correlations and another subset showing enrichment of a large positive correlation between ΔMGW and log_2_ fold change (Figure 5A). We tested whether the proportion of positive and negative correlations were equal across a range of correlation thresholds; and we observed enrichment of positive correlations with a significant trend (*P* = 0.0031) for increasing r thresholds (Figure 5B). The larger the threshold, the greater the proportion of positive correlations. To further test for a relationship between increased functional activity and greater magnitudes of ΔMGW, we compared ΔMGW percentiles for the top MPRA SNP identified in each region (see Materials and Methods). Under the null, the top SNPs from each region should exhibit a uniform distribution of ΔMGW ranks. There was significant departure from a uniform distribution based on the chi-squared goodness-of-fit test (*P* = 7.27 × 10^−4^), with an enrichment of larger ΔMGW among top MPRA-identified SNPs for each genomic region (Figure 5C).

### Fine-mapping SLE-associated genomic regions using ΔMGW prioritization identifies potentially functional SNPs

To date, more than 100 genomic loci have been associated with SLE, many which map to non-coding regions (44,64) To illustrate different scenarios based on the application of our method, we pre-selected the genomic regions containing *FAM167A-BLK*, *STAT4* and *TNIP1* for fine-mapping because these regions showed robust single-SNP associations (*P* < 5 × 10^−8^) with SLE in at least two ancestries (*FAM167A-BLK*: EA and AA; *STAT4*: EA and HA; *TNIP1*: EA and HA) and the association signals are not refined to a single SNP, due in part to strong LD. Furthermore, neither the SNPs nor their LD proxies are protein-coding variants, leaving DNA topology as a potential functional mechanism. For each region, we first describe the previous SNP association results (44) and their LD patterns, by ancestry. Each region is then summarized by its ΔMGW measures which were used in subsequent frequentist and Bayesian ΔMGW-weighted analyses. We defined successful fine-mapping as a reduction in the number of variants from among the previously observed LD blocks of association. SNPs identified by the ΔMGW-weighted analyses were subsequently investigated for existing functional evidence (see Materials and Methods).

### *FAM167A-BLK* Region

**Figure 6.**
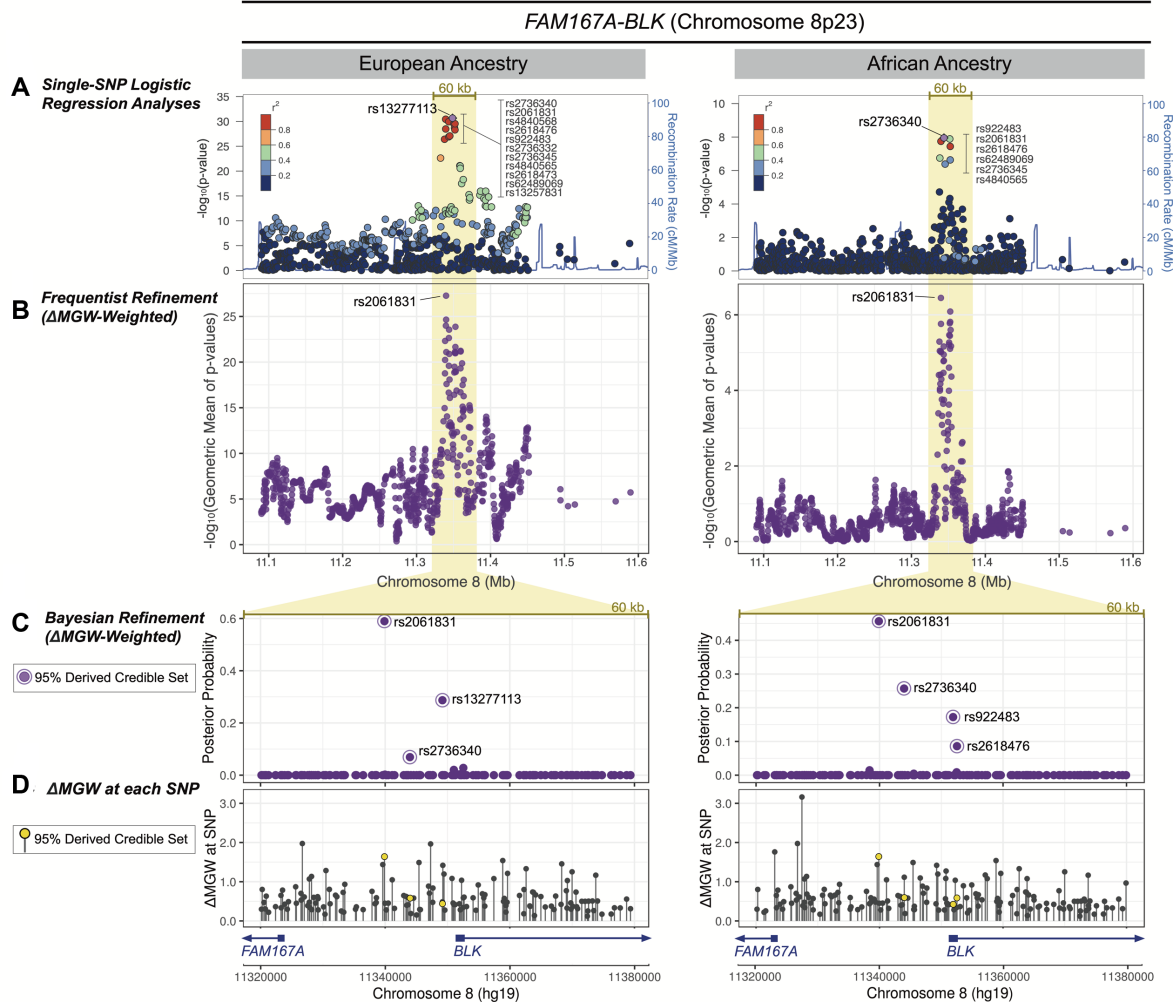
*FAM167A-BLK* ΔMGW prioritization by Frequentist and Bayesian methods in European and African Ancestries. (**A**) Genotyped SNPs that passed quality control and were within 250kb of the top single-SNP association analysis in EA and AA data. A 60 kb region capturing the primary peak of association is highlighted. In both the EA and AA data a cluster of SNPs in high LD yielded the top association signals. (**B**) Using SKAT as a ΔMGW-weighted frequentist approach, rs2061831 was sharply prioritized over SNPs in the previously identified LD blocks. While the single-SNP logistic regression analyses in (A) identified a different top SNP in the EA (rs13277113) and AA (rs2736340) data, rs2061831 was consistently prioritized as the top SNP in both the EA and AA analyses. ΔMGW-weighting did not yield spurious associations for with SNPs outside the broad 60 kb peak of association highlighted in yellow. (**C**) SNPs within the 60 kb association peak were analyzed by a Bayesian approach. The ΔMGW-weighted posterior probabilities are plotted. While the majority of SNPs yielded infinitesimal posterior probabilities, those comprising the 95% derived credible sets are labeled. Akin to the ΔMGW-weighted SKAT analyses, rs2061831 was again prioritized in both the EA and the AA data, with the largest posterior probability. (**D**) The ΔMGW is plotted for each SNP in the 60 kb region. The ΔMGW for a SNP is sequence-specific thus yielding the same values for SNPs in both the EA and AA data. Differences between the two plots result from differences in genotyped SNP lists (i.e. SNPs that are monomorphic in one population would not be plotted). SNPs identified by the derived ΔMGW-weighted credible set are plotted in yellow. While rs2061831 had a large ΔMGW, other SNPs in the region had larger magnitudes of ΔMGW but did not show evidence of SLE-association. This illustrates the importance of a two-parameter hypothesis which considers a combination of association signal and ΔMGW magnitude. Prioritized SNPs fall upstream of both *FAM167A* and *BLK*.

The SLE-associated region at 8p23 lies upstream of *FAM167A* and *BLK*, which are in a head-to-head gene orientation. In the previous (44) logistic regression analyses, the primary peak of association was captured by a 60 kb window. In EA, the most significant SNP associations mapped to a 26 kb region of 16 SNPs in high LD (r^2^>0.8); within the AA data, the top associations were refined to a smaller 14 kb window containing 7 highly correlated SNPs (Figure 6). The summary statistics for ΔMGW for SNPs in the 500 and 60 kb regions were comparable to what was observed across the genome, with only a few SNPs imposing large changes in MGW (Supplementary Table S4).

In the frequentist approach using SKAT, SNPs with the highest ΔMGW-weighted prioritizations largely followed the pattern observed in the single-SNP logistic regression analyses. That is, SNPs that were not previously associated with SLE were not prioritized solely on ΔMGW, as illustrated in the region outside of the 40 kb peak of association (Figure 6). When weighted by ΔMGW, rs2061831 was sharply prioritized in both the EA and AA analyses (Figure 6). In EA, rs2061831 was one of the 14 highly correlated SNPs identified by the single-SNP logistic regression analyses; likewise, in AA, it was also within the LD block comprising the 7 most highly associated SNPs. While the other SNPs in these LD blocks exhibited comparable SLE-association, rs2061831 had the greatest ΔMGW at 1.63 Å, prioritizing it above other SNPs in the weighted analyses. Importantly, while the single-SNP logistic regression analyses identified a different top SNP in EA (rs13277113) and AA (rs2736440) data, ΔMGW-weighting prioritized the same SNP (rs2061831), across ancestries. An unweighted SKAT prioritized the signal downstream of rs2061831, to the region where multiple SNPs from the same highly-associated LD block were included in the same 5-SNP windows (Supplementary Figure S2, Tables S5 and S6).

The ΔMGW-weighted frequentist fine-mapping evidence for rs2061831 was corroborated using the Bayesian refinement approach. In both EA and AA, the derived ΔMGW-weighted credible set placed the highest posterior probability on rs2061831 (58.9%-EA; 44.2%-AA) (Figure 6). In the unweighted (standard) Bayesian analysis, rs2061831 was included in the EA (30.6% posterior probability) and AA (20.9% posterior probability) 95% credible sets, but it was not the highest prioritized (Supplementary Tables S5 and S6). Instead, the SNPs originally identified in the ancestry-specific logistic regression analyses were given the highest posterior probability—EA: rs13277113 (49.9% posterior probability), AA: rs2736340 (33.1%). Thus, like the frequentist approach, weighting by ΔMGW resolved the signal in both EA and AA to the same SNP, rs2061831.

Using ΔMGW as a prioritization metric, rs2061831 was consistently prioritized in both EA and AA data. SNP rs2061831 has a ΔMGW of 1.63 Å, which is 2 standard deviations above the mean across dbSNP150. Notably, this SNP is a transition polymorphism (Purine/Purine), which we previously showed to have the smallest (on average) ΔMGW (Table 1, Figure 3). Considering only transition SNPs, rs2061831 is actually 4.52 standard deviations above the mean ΔMGW_transition SNPs_ (0.50 Å), indicating a considerable departure from the expected value and thus we would hypothesize a greater likelihood of functional relevance. We explored (see Methods and Materials) functional data resources for evidence of biological relevance, in comparison to the top SNP signals from the single-SNP analyses (rs13277113 in EA; and rs2736440 in AA). All three SNPs are in high LD (*R*^2^ > 0.95) with one another in both EUR and AFR 1000 genomes data. Thus, it is not surprising that all three SNPs yielded similar eQTL results via GTEx (data not shown). Despite the high LD, these three SNPs are physically separated by several kilobases. Both rs2061831 and rs13277113 mapped to Candidate *cis*-Regulatory Elements (cCRE) (accession numbers: EH38E2610769 and EH38E2610775, respectively). Of note, the cCRE mapping to rs2061831 showed high CTCF activity with supportive TF binding (Supplementary Figure S3). Review of the 3D-genome browser yielded a larger number of long-range chromatin interactions in monocytes, B-Cells, and CD4 cells for rs2061831, in comparison to rs13277113 and rs2736440 (Supplementary Figure S4). In the *FAM167A-BLK* region, ΔMGW-weighting successfully differentiated among highly-correlated SNPs and prioritized rs2061831, a SNP within a potentially important regulatory region as documented by independent data.

### *STAT4* Region

**Figure 7.**
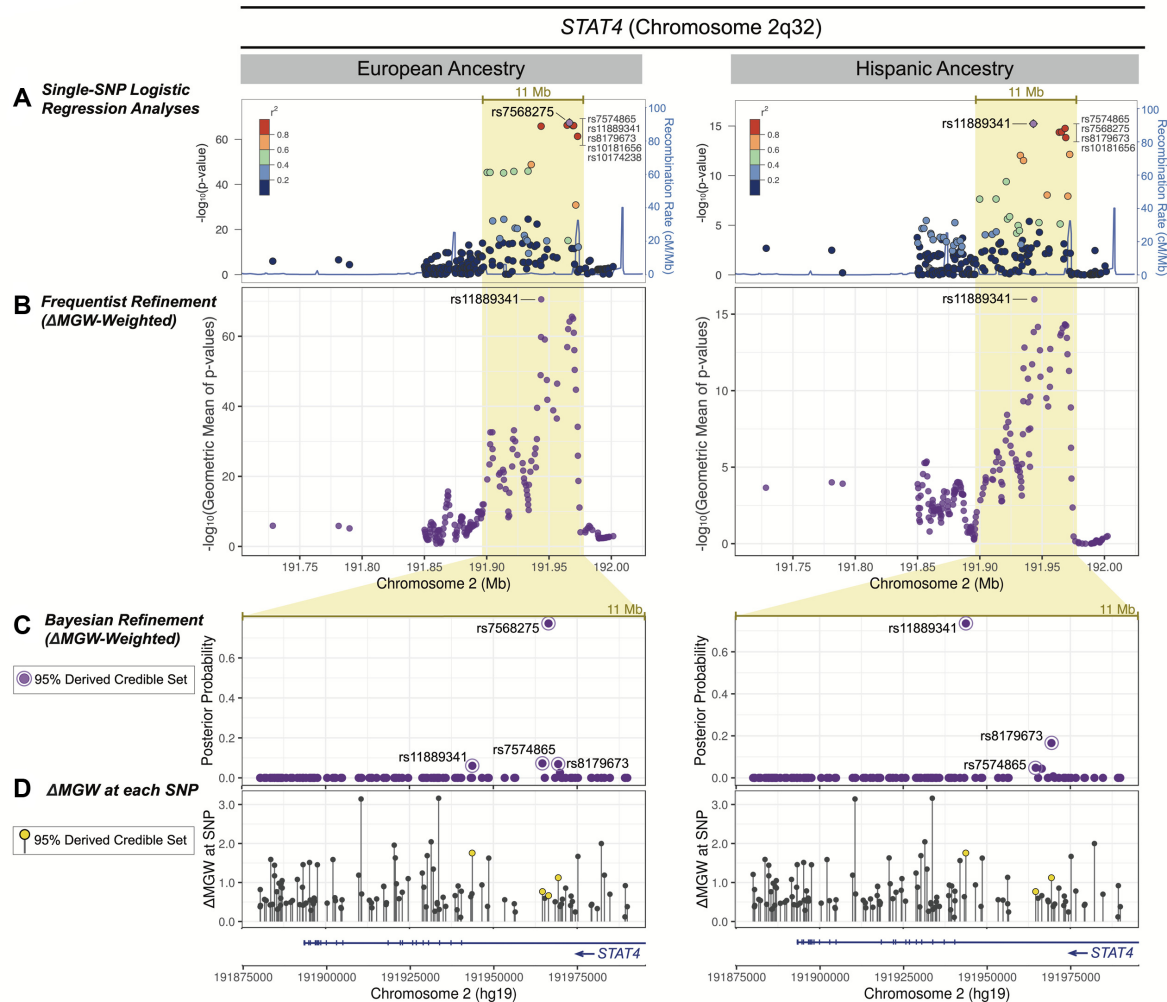
*STAT4* ΔMGW prioritization by Frequentist and Bayesian methods in European and Hispanic Ancestries. (**A**) Regional association plots in EA and HA for genotyped SNPs that passed quality control and were within 250 kb of the top single-SNP association analysis in *STAT4*. Within the broad 11 Mb peak of association (highlighted in yellow), a cluster of SNPs in high LD yielded the top association values. (**B**) SNP refinement using SKAT with a ΔMGW-weighted approach sharply prioritizes rs11889341 in both EA and HA data. In the EA data, the ΔMGW-weighting shifted the top signal to rs1188931, whereas in the HA data, it accentuated the existing signal, above other SNPs. (**C**) For the highlighted 11 Mb region, SNP posterior probabilities are plotted for the derived, ΔMGW-weighted Bayesian analysis. While the frequentist MGW-weighted approach prioritized the same SNP (rs1188931) in both ancestries, this was not observed in the Bayesian approach. In the EA data, the Bayes factor for rs7568275 (BF = 2.20 × 10^64^) was at such a large magnitude, that it was largely unaffected by ΔMGW-weighting. However, rs1188931 still entered the 95% derived credible set, albeit with a much smaller posterior probability (6.03%) compared to rs7568275 (77.25%). In the HA data, ΔMGW-weighting increased the signal for rs1188931. (**D**) The ΔMGW for SNPs within the 11 Mb region. SNPs that were identified by the derived ΔMGW-weighted credible set are plotted in yellow. Again, the analytic approaches consider SNPs in the context of a two-parameter hypothesis, evaluating SNPs for a combination of association signal and magnitude of ΔMGW. Hence, the prioritized SNPs (yellow) are not necessarily the SNPs with the largest ΔMGW in the region. Prioritized SNPs occur within an intron of *STAT4*.

The single-SNP SLE associations at 2q32 span the *STAT4* gene (Figure 7). SNP associations reached genome significance in the EA and HA data, with the strongest signals within intronic regions (44). In both ancestries, the primary peak of association was captured by a broad 110 kb window. The strongest associations in the EA data (*P*-values < 1 × 10^−62^) mapped to six SNPs in high LD, spanning 29 kb. Five of these SNPs also comprised the LD block of strongest associations in the HA data (*P* < 1 × 10^−13^), in a slightly narrower 26 kb region.

The mean ΔMGW for SNPs in this region was 0.72 Å in EA and 0.73 Å in HA and both cohorts had a median ΔMGW of 0.56 Å. While these average ΔMGW were slightly higher than what was observed across the entire bi-allelic dbSNP dataset (mean = 0.68 Å), the EA and HA medians were of the same magnitude (dbSNP ΔMGW median = 0.56). The ΔMGW for SNPs within the 110 kb association window exhibited similar means as the 500 kb region (Supplementary Table S7).

We again applied the two ΔMGW-weighted approaches using SKAT and Bayesian credible sets in the region. In EA, the ΔMGW-weighted SKAT analyses shifted the top signal upstream to rs11889341, which markedly increased its priority (Figure 7; Supplementary Figure S5). This SNP was one of the top six SNPs in the single-SNP association LD-block. While it and the other five SNPs were all significantly associated with SLE, rs11889341 had the greatest ΔMGW at 1.75 Å, which prioritized it over the other SNPs in the LD block; the remaining SNPs had ΔMGW values ranging from 0.31–1.12 Å. In HA, weighting by ΔMGW in the SKAT analysis also prioritized rs11889341 as the top SNP. This SNP was previously identified with the best *P*-value in the single-SNP association analysis, but in the ΔMGW-weighted approach, its prioritization distinctly increased relative to the other SNPs in the LD block.

In the Bayesian analysis, rs11889341 was included in the EA and HA derived ΔMGW-weighted 95% credible sets. In EA, rs11889341 was not in the unweighted 95% credible set but inclusion of ΔMGW increased its posterior probability from 2.4% to 6.0% (Supplementary Table S8). In EA, rs7568275 yielded the strongest signal in both the unweighted (81.0% posterior probability) and derived ΔMGW-weighted (77.3% posterior probability) credible sets (Supplementary Table S8). This is important to note, as rs7568275 had a much smaller ΔMGW (0.66 Å) than rs11889341 (1.75 Å). This provided an example where the magnitude of the Bayes factor was so large (2.20 × 10^64^), that the influence of ΔMGW was largely diminished in the analysis. However, despite the predominant rs7568275 signal, the derived credible set still detected rs11889341, the SNP identified by the ΔMGW-weighted SKAT approach. In the HA data, rs11889341 yielded the largest posterior probability in the ΔMGW-weighted derived credible set. This SNP also had the largest posterior probability in the unweighted credible set. Unlike the EA analysis, where the magnitude of the Bayes factor dominated the impact of the ΔMGW-weighting, in the HA data, the ΔMGW strongly increased the posterior probability of rs11889341 from 58.6% to 73.5%. This limited the derived 95% credible set to only 3 SNPs: rs11889341 (73.5%), rs8179673 (16.6%) and rs7574865 (4.8%) (Supplementary Table S9).

In ΔMGW-weighted analyses, rs11889341 was sharply prioritized over other SNPs in the LD block, with an exception in the EA ΔMGW-weighted derived credible set, where the high magnitude of the Bayes factor for rs7568275 (2.20 × 10^64^) over other SNPs (bf ≤ 1.79 × 10^63^) largely negated the impact of ΔMGW in this analysis. Considering the evidence for rs11889341 in the other three analyses, due to its strong combination of SLE association and ΔMGW, we would hypothesize that rs11889341 would be a candidate functional polymorphism. Like rs2061831 in *FAM167A-BLK*, rs11889341 is also a transition SNP (purine/purine). While transition SNPs are more frequent across the genome (previously shown in Supplementary Table S1), there are few transition SNPs (+/- 4 nucleotides) that yield such a high ΔMGW (mean ΔMGW for transition SNPs = 0.50 Å). Evaluation of publicly available functional datasets yielded limited information for both rs7568275 and rs11889341. Neither of these SNPs were identified as eQTLs in GTEx nor were they within Candidate Cis-Regulatory regions (cCREs). Furthermore, neither variant was shown with long range chromatin interactions in the in the currently available HI-C data via the 3D genome browser. Despite the lack of functional information from these publicly available databases, functional information is available via a 2018 study, where transancestral mapping identified rs11889341 with strong association with SLE (65). This study focused on the *STAT1-STAT4* region and found rs11889341 was associated with *STAT1* expression in B-cells through increased binding of the transcription factor, HMGA1 (65). Given the relationship between transcription factor binding and DNA topology (20,31,32,66,67), we hypothesize that the identified functional activity of rs11889341 (via HMGA1 binding) may be mediated by the large MGW change imposed by the SNP’s alleles.

### *TNIP1* Region

**Figure 8.**
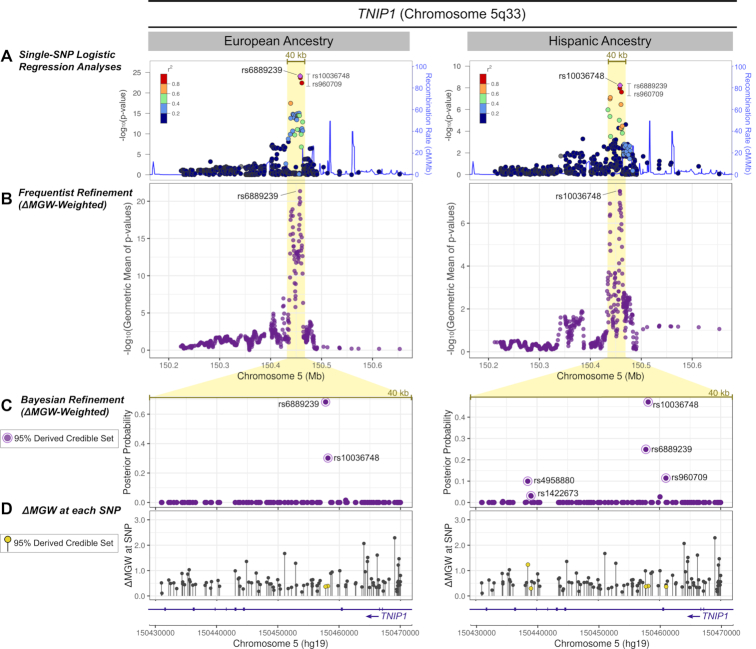
*TNIP1* ΔMGW prioritization by Frequentist and Bayesian methods in European and Hispanic Ancestries. (**A**) Genotyped SNPs within 250 kb of the top single-SNP association analysis are shown for EA and HA. The 40 kb region that captures the primary peak of association is highlighted in yellow. In EA and HA, the same three SNPs (rs10036748, rs6889239, and rs960709) show the highest association values and are all in high LD. In EA data, rs6889239 has the best *P*-value and rs10036748 yields the best *P*-value in HA data. (**B**) Analyzing the region with SKAT in a ΔMGW-weighted approach. In this region, for these SNPs, including ΔMGW did not provide differential prioritization, rs6889239 remained the top signal for EA and rs10036748 for HA. (**C**) For each SNP in the 40 kb region, the posterior probabilities are plotted for the derived, ΔMGW-weighted Bayesian analysis. The weighted Bayesian analysis did not alter the relative signals observed in the single-SNP logistic regression analyses. In the EA data, rs6889239 yielded the largest posterior probability in EA and rs10036748 remained the top signal in HA data. (**D**) The ΔMGW is plotted for each genotyped SNP that passed quality control measures. SNPs that were identified by the derived ΔMGW-weighted credible set are plotted in yellow. These prioritized SNPs have comparatively low magnitudes of ΔMGW, indicating that the driving factor of these SNP prioritizations stemmed from their SLE associations and not their magnitude of ΔMGW.

Previous single-SNP association analyses (44) identified genome-wide significant findings (*P* < 5 × 10^−8^) in EA and HA data at 5q33 (Figure 8). The peak of SLE association is captured by a 40 kb window which encompasses most of the *TNIP1* gene. In the EA data, the top associations mapped to three SNPs (rs960709, rs10036748, rs6889239) in high LD, spanning 3 kb of a *TNIP1* intron. These three SNPs are also encompassed by the associated LD block in the HA data, where four, highly correlated SNPs (rs1422673, rs960709, rs10036748 and rs6889239) yielded *P*-values < 5 × 10^−8^. As completed in the *FAM167A-BLK* and *STAT4* regions, we again applied ΔMGW-weighted fine-mapping strategies to prioritize these non-coding SLE-associated SNPs.

In the *TNIP1* region, the lists of high-quality genotyped SNPs were largely the same between the EA and HA datasets. Consequently, the statistics for ΔMGW in this region were very similar between the two cohorts. Across the 500 kb window of high quality SNPs, the mean ΔMGW was 0.67 Å (median = 0.55 Å) in both EA and HA (Supplementary Table S10). These values were slightly lower than the observed mean for bi-allelic SNPs from dbSNP (Table 1).

The SKAT analyses yielded similar results between the EA and HA data. The ΔMGW-weighted analyses did not effectively prioritize or refine the SNP signal. Unlike *FAM167A-BLK* and *STAT4*, ΔMGW-weighting did not resolve the top signal to the same SNP in both ancestries. Instead, in *TNIP1*, the top SNPs in the ΔMGW-weighted analyses for EA (rs6889239) and HA (rs10036748) were the same as those identified in the single-SNP logistic regression analysis (Figure 8; Supplementary Figure S6). In this region ΔMGW-weighting actually dampened the signal because the SNPs with the greatest SLE association values had low magnitudes of ΔMGW (ranging from 0.31 to 0.37 Å). This pattern was also observed in the Bayesian approach, where SNPs with the highest posterior probabilities in the derived credible sets exhibited lower posterior probabilities than in the unweighted credible set (Figure 8; Supplementary Tables S11–12), again due to the low magnitudes of ΔMGW for top-associated SNPs.

## DISCUSSION

Sequence-dependent DNA topology could provide important functional context for associations, especially for polymorphisms in non-coding regions and/or in regions not covered by currently available functional databases. We explored ΔMGW, a specific sequence-dependent measure of DNA topology, as a weighting metric in fine-mapping analyses. In a sample of 300k SNPs, Wang *et al.* previously found that MGW-preserving SNPs are more common (42). Here, we built upon these findings through a full census of bi-allelic SNPs (*n* = 199 038 197) across the genome. We showed the observed genomic ΔMGW was lower than the complete ΔMGW sample space. These findings were consistent with the relative frequencies of transversion (∼33%) and transition (∼66%) mutations in the human genome (61,62). We hypothesized that phenotypically-associated SNPs with large ΔMGW would be more likely to impose functional consequences; and thus, proposed ΔMGW as a prioritization metric in fine-mapping studies. To test for and delineate the relationship between ΔMGW and allele-specific activity, we analyzed data from an existing MPRA study (43). We observed significant enrichment for strong positive correlations, illustrating the presence of a relationship between ΔMGW and allele-specific activity. We do not posit that this relationship holds for every SNP but that it will be a useful additional prioritization metric. In fact, consistent with our hypothesis, a large positive correlation was not observed in every genomic region, but across the regions studied, there was an enrichment. Further, we observed enrichment for the largest (regional) ΔMGW among SNPs that exhibited the greatest magnitude of log_2_ fold change via the MPRA. While there is evidence that changes in MGW can affect aspects that may go undetected by such a reporter assay (e.g. methylation, chromatin remodeling), the observed patterns importantly document a potential role between ΔMGW and functionality.

To illustrate an application of our approach, we applied two ΔMGW-weighted fine-mapping approaches, across three genomic regions (*FAM167A-BLK*, *STAT4*, and *TNIP1*) with well-established SLE associations. In *FAM167A-BLK* and *STAT4*, we successfully reduced the number of potential functional SNPs to a single, transancestral SNP in each region. For both of these ΔMGW-identified SNPs, we identified external evidence supporting their functional roles.

There are several advantages to using sequence dependent topology, such as ΔMGW, as a weighting metric in fine-mapping studies. For one, it is an intrinsic variable, inherent to the genetic sequence surrounding the polymorphism; thus, it is not reliant on external databases which may offer limited information for the SNPs of interest (database bias). As an intrinsic variable it is also not ancestry specific, tissue specific, or sample size dependent. Limitations in external (non-intrinsic) data may down-weight potentially causal SNPs due to a lack of available functional data. While publicly available functional resources continue to expand, these challenges remain, especially for rare or novel variants. This is particularly relevant for diverse study populations where annotation resources based on European data may offer limited information for regions of interest (14). For example, Sherman *et al.* presented deep sequencing data in 910 individuals of African descent and found over 296 million base pairs which were absent in the human reference genome (15). While a SNP’s functional relevance can be supported by publicly-available resources, a lack of information does not necessarily indicate a variant's lack of function. This was illustrated by rs11889341 in *STAT4*, which lacked functional information from public resources (GTEx, ENCODE, 3D-genome browser) (57,58,60), but in a targeted functional study, rs11889341 was correlated with gene expression and binding of the transcription factor HMGA1 (65). We identified rs11889341 using ΔMGW as the prioritizing variable. Thus, prioritizing SNPs by a factor intrinsic to DNA may help alleviate some bias that would otherwise be introduced by missing data from publicly available functional datasets.

A second benefit of using DNA topology in fine-mapping is that DNA topology (e.g. MGW) can potentially impact an array of biological functions such as transcription factor binding, chromatin remodeling, or methylation (20,21,23,26,31,32,36). By using ΔMGW (e.g. instead of weighting by specific MGW patterns for a particular transcription factor), the approaches in this manuscript are agnostic to the mechanism of function imposed by MGW. We posit this as beneficial, as it does not limit functional information to a single biological mechanism. This may be especially beneficial when the relationship between phenotype and biological mechanism is unknown. While functional work in *STAT4* showed that rs11889341 altered HMGA1 binding, functional work is still needed to evaluate the rs2061831 genotype in *FAM167A-BLK*. Here, the biological implications of rs2061831 could involve transcription factor binding, and/or, given its apparent location within a long-range chromatin interaction hotspot (Supplementary Figure S4), chromatin organization. Considering the strong trans-ancestral signal of rs2061831 across EA and AA, further functional work should explore whether this SNP acts through an independent functional mechanism or through interactions with other variants in the region (e.g. within the context of sequence-dependent structural motifs), such as the insertion-deletion identified in a study of ATAC-seq data in 100 individuals of British Ancestry (68). Leveraging changes in DNA topology can identify potentially causal polymorphisms and also generate specific hypotheses for functional follow-up studies.

Another advantage to using local DNA topology in fine-mapping studies is its consistency of information across ancestries. Assuming identical flanking sequences (e.g. no genomic variant within ±4 bases of the SNP), a SNP’s impact on intrinsic DNA topology is consistent across ancestries, highlighting the potential utility of DNA topology as a means of resolving association signals across ancestries. Here, we showed that ΔMGW-weighted analyses of *FAM167A*-*BLK* and *STAT4* resolved the association signal to the same SNP in each ancestry via the frequentist approach, followed by largely corroborating evidence via the derived credible sets in the Bayesian approach. Notably, rs2061831 was not the top-associated SNP in either the ancestry-specific analyses; however, it was previously identified via the SLE Immunochip trans-ancestral meta-analysis, where combining association signals across ancestries identified it as the top SNP (44).

### Limitations and future work

There are several considerations and limitations to using sequence-dependent topology as a weighting metric in fine-mapping analyses. Notably, some of these limitations could result in inconclusive and/or insignificant results, as observed in the *TNIP1* region. First, the functional variants may not have been genotyped in the study. Analyses that utilize SNP-specific weights decouple associations from LD. Thus, a weighted metric performs best when the functional SNP is included in the analysis set. For this reason, we propose application of this prioritization technique when there is high confidence that the functional variants have been included through dense genotyping or sequencing. We note this limitation exists for any statistical association method.

Second, DNA topology may not be the mechanism impacting phenotype at a particular locus. While sequence dependent DNA topology can influence a number of functional factors (18,21,23,24,32), it is not the only source of biological interactions and could be irrelevant for a specific phenotype or genomic region. Thus, when using DNA topology, such as ΔMGW, in fine-mapping studies, analyses should be considered in the form of a two-parameter hypothesis—a combination of association signal and ΔMGW. For example, in both the *FAM167A-BLK* and *STAT4* regions, the highest prioritized SNPs, rs2061831 and rs11889341, did not have the largest magnitude of ΔMGW in the regions (Figures 6 and 7). Instead, these two SNPs were prioritized by their combined signals of SLE-association and ΔMGW.

Third, we placed greater weights on SNPs with larger magnitudes of change on DNA topology. We recognize that even small changes could yield functional consequences. While we previously described benefits to using an agnostic measure of MGW (ΔMGW magnitude), future studies could also explore weighting SNPs by particular topological profiles (e.g., those matching binding site profiles). For instance, our *TNIP1* analyses did not show strong signals when weighting by the magnitude of ΔMGW, but this does not definitively rule out MGW as a functional mechanism (e.g. driven by pattern, not magnitude). The focus on MGW was motivated by the breadth of study on MGW and function (18,20,32,34,36). So while this manuscript considered a single parameter, ΔMGW, we are currently expanding to incorporate additional features (e.g. helix twist, roll) through multivariate approaches that account for the correlation structure (dependencies) among spatial measures.

Fourth, there are some limitations to the methods implemented through ΔMGW-weighted SKAT and the derived credible sets approach. Here, we assumed that the majority of variants in the region are non-causal, which is why we selected SKAT over a combined burden test. However, we note that the results from SKAT and SKAT-O were largely similar. Similarly, in case of the applied Bayesian approach, a limitation is its assumption that a single causal SNP exists in a region (54). In the EA *STAT4* data, the magnitudes of the Bayes factors were so large that weighting by ΔMGW yielded minimal impact. Future work should consider approaches to scale weighting schemes by a constant derived from the magnitude of signal across a genomic region. In the SKAT approach, for the sliding analysis window, we used five SNPs, which should yield a region that is neither too wide nor too unstable. Additional testing could potentially improve optimization of parameters for this analysis. Furthermore, we emphasize that our evaluation of the SKAT results by summarizing each SNP as the geometric mean of SKAT-analysis *P*-values should be regarded as a metric for prioritizing SNPs, not an association analyses, as these values do not have the statistical properties of a *P*-value. Overall, these limitations should be carefully considered when applying these specific methods; but they also highlight opportunities to further explore the relationship between sequence-dependent DNA topology and phenotype associations.

We note that, at present there are limited options for incorporating continuous values for SNP-specific weights. In our frequentist approach, we utilized SKAT for its flexibility in accepting user-specified weights. In our Bayesian method we used derived credible sets based on *ad hoc* weighting of the posterior probabilities by ΔMGW. While there are existing Bayesian methods that can incorporate functional annotations, we note these require binary categorical values and assume agnostic application of annotations and thus adjust prioritization based on enrichment of the annotation in associations (55,69). That is, these methods do not assume the existence of an *a priori* hypothesis for inclusion of user-specified weights. Under our two-parameter hypothesis, we posit that at a locus, within a set of comparably associated variants, only one or two (not the majority) may show large magnitude of ΔMGW. For this reason, we did not apply these existing methods within this manuscript. While we showed success with the two methods applied here, we also propose incorporating intrinsic DNA topology (e.g. ΔMGW), as an annotation resource in other statistical methods as they develop.

In summary, weighting SNP associations by functional data can greatly improve identification of potentially causal SNPs; however, existing annotation resources can negatively affect these outcomes when SNP information is unavailable in public datasets, especially in novel genomic regions (8,10,11,14). In this study, we presented and tested sequence-dependent DNA topology as a novel annotation source for genetic fine-mapping studies. As an intrinsic property, sequence-dependent DNA shape alleviates many of the challenges imposed by external data resources; and it provides potential functional (testable) context for associations (e.g. topological disruption for protein binding). Using ΔMGW in weighted analyses, we successfully prioritized functional SNPs in two SLE-associated regions with high LD. Likewise, as an annotation resource, sequence-dependent DNA topology, such as ΔMGW, is readily applicable in any fine-mapping methods that can incorporate continuous values for SNP weights. Altogether, this manuscript presents methods that are immediately applicable to existing genetic data, and it illustrates how sequence-dependent DNA topology can be used as a paradigm to investigate and understand genetic associations in fine-mapping studies.

## DATA AVAILABILITY

Data used within this manuscript was obtained from publicly available resources (with relevant links noted in-text) and previously published references (43) and (44).

## Supplementary Material

gkaa877_Supplemental_Files

## References

[1] MacArthur J., Bowler E., Cerezo M., Gil L., Hall P., Hastings E., Junkins H., McMahon A., Milano A., Morales J. et al. The new NHGRI-EBI Catalog of published genome-wide association studies (GWAS Catalog). Nucleic Acids Res. 2017; 45:D896–D901. 27899670 10.1093/nar/gkw1133 PMC5210590

[2] Visscher P.M., Brown M.A., McCarthy M.I., Yang J. Five years of GWAS discovery. Am. J. Hum. Genet. 2012; 90:7–24. 22243964 10.1016/j.ajhg.2011.11.029 PMC3257326

[3] Visscher P.M., Wray N.R., Zhang Q., Sklar P., McCarthy M.I., Brown M.A., Yang J. 10 Years of GWAS discovery: biology, function, and translation. Am. J. Hum. Genet. 2017; 101:5–22. 28686856 10.1016/j.ajhg.2017.06.005 PMC5501872

[4] McCarthy M.I., Abecasis G.R., Cardon L.R., Goldstein D.B., Little J., Ioannidis J.P.A., Hirschhorn J.N. Genome-wide association studies for complex traits: consensus, uncertainty and challenges. Nat. Rev. Genet. 2008; 9:356–369. 18398418 10.1038/nrg2344

[5] Manolio T.A., Collins F.S., Cox N.J., Goldstein D.B., Hindorff L.A., Hunter D.J., McCarthy M.I., Ramos E.M., Cardon L.R., Chakravarti A. et al. Finding the missing heritability of complex diseases. Nature. 2009; 461:747–753. 19812666 10.1038/nature08494 PMC2831613

[6] Pasaniuc B., Price A.L. Dissecting the genetics of complex traits using summary association statistics. Nat. Rev. Genet. 2017; 18:117–127. 27840428 10.1038/nrg.2016.142 PMC5449190

[7] Farh K.K.-H., Marson A., Zhu J., Kleinewietfeld M., Housley W.J., Beik S., Shoresh N., Whitton H., Ryan R.J.H., Shishkin A.A. et al. Genetic and epigenetic fine mapping of causal autoimmune disease variants. Nature. 2015; 518:337–343. 25363779 10.1038/nature13835 PMC4336207

[8] Gomez-Cabrero D., Abugessaisa I., Maier D., Teschendorff A., Merkenschlager M., Gisel A., Ballestar E., Bongcam-Rudloff E., Conesa A., Tegnér J. Data integration in the era of omics: current and future challenges. BMC Syst. Biol. 2014; 8:I1. 10.1186/1752-0509-8-S2-I1 PMC4101704 25032990

[9] Faye L.L., Machiela M.J., Kraft P., Bull S.B., Sun L. Re-Ranking sequencing variants in the Post-GWAS Era for accurate causal variant identification. PLoS Genet. 2013; 9:e1003609. 23950724 10.1371/journal.pgen.1003609 PMC3738448

[10] Kichaev G., Yang W.-Y., Lindstrom S., Hormozdiari F., Eskin E., Price A.L., Kraft P., Pasaniuc B. Integrating functional data to prioritize causal variants in statistical fine-mapping studies. PLoS Genet. 2014; 10:e1004722. 25357204 10.1371/journal.pgen.1004722 PMC4214605

[11] Xu Z., Taylor J.A. SNPinfo: integrating GWAS and candidate gene information into functional SNP selection for genetic association studies. Nucleic Acids Res. 2009; 37:W600–W605. 19417063 10.1093/nar/gkp290 PMC2703930

[12] Lee S., Wu M.C., Lin X. Optimal tests for rare variant effects in sequencing association studies. Biostatistics. 2012; 13:762–775. 22699862 10.1093/biostatistics/kxs014 PMC3440237

[13] Nicolae D.L., Gamazon E., Zhang W., Duan S., Dolan M.E., Cox N.J. Trait-Associated SNPs Are More Likely to Be eQTLs: Annotation to Enhance Discovery from GWAS. PLoS Genet. 2010; 6:e1000888. 20369019 10.1371/journal.pgen.1000888 PMC2848547

[14] Kessler M.D., Yerges-Armstrong L., Taub M.A., Shetty A.C., Maloney K., Jeng L.J.B., Ruczinski I., Levin A.M., Williams L.K., Beaty T.H. et al. Challenges and disparities in the application of personalized genomic medicine to populations with African ancestry. Nat. Commun. 2016; 7:12521. 27725664 10.1038/ncomms12521 PMC5062569

[15] Sherman R.M., Forman J., Antonescu V., Puiu D., Daya M., Rafaels N., Boorgula M.P., Chavan S., Vergara C., Ortega V.E. et al. Assembly of a pan-genome from deep sequencing of 910 humans of African descent. Nat. Genet. 2019; 51:30–35. 30455414 10.1038/s41588-018-0273-y PMC6309586

[16] Need A.C., Goldstein D.B. Next generation disparities in human genomics: concerns and remedies. Trends Genet. TIG. 2009; 25:489–494. 19836853 10.1016/j.tig.2009.09.012

[17] Manrai A.K., Funke B.H., Rehm H.L., Olesen M.S., Maron B.A., Szolovits P., Margulies D.M., Loscalzo J., Kohane I.S. Genetic misdiagnoses and the potential for health disparities. N. Engl. J. Med. 2016; 375:655–665. 27532831 10.1056/NEJMsa1507092 PMC5292722

[18] Privalov P.L., Dragan A.I., Crane-Robinson C., Breslauer K.J., Remeta D.P., Minetti C.A.S.A. What drives proteins into the major or minor grooves of DNA?. J. Mol. Biol. 2007; 365:1–9. 17055530 10.1016/j.jmb.2006.09.059 PMC1934558

[19] Yakovchuk P., Protozanova E., Frank-Kamenetskii M.D. Base-stacking and base-pairing contributions into thermal stability of the DNA double helix. Nucleic Acids Res. 2006; 34:564–574. 16449200 10.1093/nar/gkj454 PMC1360284

[20] Yang L., Orenstein Y., Jolma A., Yin Y., Taipale J., Shamir R., Rohs R. Transcription factor family-specific DNA shape readout revealed by quantitative specificity models. Mol. Syst. Biol. 2017; 13:910. 28167566 10.15252/msb.20167238 PMC5327724

[21] Duan C., Huan Q., Chen X., Wu S., Carey L.B., He X., Qian W. Reduced intrinsic DNA curvature leads to increased mutation rate. Genome Biol. 2018; 19:132. 30217230 10.1186/s13059-018-1525-y PMC6138893

[22] Sati S., Cavalli G. Chromosome conformation capture technologies and their impact in understanding genome function. Chromosoma. 2017; 126:33–44. 27130552 10.1007/s00412-016-0593-6

[23] Lazarovici A., Zhou T., Shafer A., Dantas Machado A.C., Riley T.R., Sandstrom R., Sabo P.J., Lu Y., Rohs R., Stamatoyannopoulos J.A. et al. Probing DNA shape and methylation state on a genomic scale with DNase I. Proc. Natl. Acad. Sci. U.S.A. 2013; 110:6376–6381. 23576721 10.1073/pnas.1216822110 PMC3631675

[24] Abe N., Dror I., Yang L., Slattery M., Zhou T., Bussemaker H.J., Rohs R., Mann R.S. Deconvolving the recognition of DNA shape from sequence. Cell. 2015; 161:307–318. 25843630 10.1016/j.cell.2015.02.008 PMC4422406

[25] Bansal M., Kumar A., Yella V.R. Role of DNA sequence based structural features of promoters in transcription initiation and gene expression. Curr. Opin. Struct. Biol. 2014; 25:77–85. 24503515 10.1016/j.sbi.2014.01.007

[26] Parker S., Tullius T.D. DNA shape, genetic codes, and evolution. Curr. Opin. Struct. Biol. 2011; 21:342–347. 21439813 10.1016/j.sbi.2011.03.002 PMC3112471

[27] Olson W.K., Bansal M., Burley S.K., Dickerson R.E., Gerstein M., Harvey S.C., Heinemann U., Lu X.-J., Neidle S., Shakked Z. et al. A standard reference frame for the description of nucleic acid Base-pair geometry. J. Mol. Biol. 2001; 313:229–237. 11601858 10.1006/jmbi.2001.4987

[28] Lu X.-J., Olson W.K. Resolving the discrepancies among nucleic acid conformational analyses11Edited by I. Tinoco. J. Mol. Biol. 1999; 285:1563–1575. 9917397 10.1006/jmbi.1998.2390

[29] Dickerson R.E. Definitions and nomenclature of nucleic acid structure components. Nucleic Acids Res. 1989; 17:1797–1803. 2928107 10.1093/nar/17.5.1797 PMC317523

[30] Rohs R., West S.M., Sosinsky A., Liu P., Mann R.S., Honig B. The role of DNA shape in protein-DNA recognition. Nature. 2009; 461:1248–1253. 19865164 10.1038/nature08473 PMC2793086

[31] Meysman P., Marchal K., Engelen K. DNA structural properties in the classification of genomic transcription regulation elements. Bioinforma. Biol. Insights. 2012; 6:155–168. 10.4137/BBI.S9426 PMC3399529 22837642

[32] Stella S., Cascio D., Johnson R.C. The shape of the DNA minor groove directs binding by the DNA-bending protein Fis. Genes Dev. 2010; 24:814–826. 20395367 10.1101/gad.1900610 PMC2854395

[33] Irobalieva R.N., Fogg J.M., Catanese D.J., Catanese D.J., Sutthibutpong T., Chen M., Barker A.K., Ludtke S.J., Harris S.A., Schmid M.F. et al. Structural diversity of supercoiled DNA. Nat. Commun. 2015; 6:8440. 26455586 10.1038/ncomms9440 PMC4608029

[34] Morgunova E., Yin Y., Jolma A., Dave K., Schmierer B., Popov A., Eremina N., Nilsson L., Taipale J. Structural insights into the DNA-binding specificity of E2F family transcription factors. Nat. Commun. 2015; 6:10050. 26632596 10.1038/ncomms10050 PMC4686757

[35] Ngo T.T.M., Zhang Q., Zhou R., Yodh J.G., Ha T. Asymmetric unwrapping of nucleosomes under tension directed by DNA local flexibility. Cell. 2015; 160:1135–1144. 25768909 10.1016/j.cell.2015.02.001 PMC4409768

[36] Perino M., van Mierlo G., Karemaker I.D., van Genesen S., Vermeulen M., Marks H., van Heeringen S.J., Veenstra G.J.C. MTF2 recruits polycomb repressive complex 2 by helical-shape-selective DNA binding. Nat. Genet. 2018; 50:1002–1010. 29808031 10.1038/s41588-018-0134-8

[37] Chen C., Pettitt B.M. DNA shape versus sequence variations in the protein binding process. Biophys. J. 2016; 110:534–544. 26840719 10.1016/j.bpj.2015.11.3527 PMC4744204

[38] Shepherd J.W., Greenall R.J., Probert M.I.J., Noy A., Leake M.C. The emergence of sequence-dependent structural motifs in stretched, torsionally constrained DNA. Nucleic Acids Res. 2020; 48:1748–1763. 31930331 10.1093/nar/gkz1227 PMC7038985

[39] Chiu T.-P., Comoglio F., Zhou T., Yang L., Paro R., Rohs R. DNAshapeR: an R/Bioconductor package for DNA shape prediction and feature encoding. Bioinformatics. 2016; 32:1211–1213. 26668005 10.1093/bioinformatics/btv735 PMC4824130

[40] Zhou T., Shen N., Yang L., Abe N., Horton J., Mann R.S., Bussemaker H.J., Gordân R., Rohs R. Quantitative modeling of transcription factor binding specificities using DNA shape. Proc. Natl. Acad. Sci. U.S.A. 2015; 112:4654–4659. 25775564 10.1073/pnas.1422023112 PMC4403198

[41] Duzdevich D., Redding S., Greene E. DNA dynamics and single-molecule biology. Chem. Rev. 2014; 114:3072–3086. 24400809 10.1021/cr4004117 PMC3968236

[42] Wang X., Zhou T., Wunderlich Z., Maurano M.T., DePace A.H., Nuzhdin S.V., Rohs R. Analysis of genetic variation indicates DNA shape involvement in purifying selection. Mol. Biol. Evol. 2018; 35:1958–1967. 29850830 10.1093/molbev/msy099 PMC6063282

[43] Tewhey R., Kotliar D., Park D.S., Liu B., Winnicki S., Reilly S.K., Andersen K.G., Mikkelsen T.S., Lander E.S., Schaffner S.F. et al. Direct identification of hundreds of expression-modulating variants using a multiplexed reporter assay. Cell. 2016; 165:1519–1529. 27259153 10.1016/j.cell.2016.04.027 PMC4957403

[44] Langefeld C.D., Ainsworth H.C., Graham D.S.C., Kelly J.A., Comeau M.E., Marion M.C., Howard T.D., Ramos P.S., Croker J.A., Morris D.L. et al. Transancestral mapping and genetic load in systemic lupus erythematosus. Nat. Commun. 2017; 8:16021. 28714469 10.1038/ncomms16021 PMC5520018

[45] van Dijk M., Bonvin A.M.J.J. 3D-DART: a DNA structure modelling server. Nucleic Acids Res. 2009; 37:W235–W239. 19417072 10.1093/nar/gkp287 PMC2703913

[46] Pettersen E.F., Goddard T.D., Huang C.C., Couch G.S., Greenblatt D.M., Meng E.C., Ferrin T.E. UCSF chimera–a visualization system for exploratory research and analysis. J. Comput. Chem. 2004; 25:1605–1612. 15264254 10.1002/jcc.20084

[47] Haeussler M., Zweig A.S., Tyner C., Speir M.L., Rosenbloom K.R., Raney B.J., Lee C.M., Lee B.T., Hinrichs A.S., Gonzalez J.N. et al. The UCSC Genome Browser database: 2019 update. Nucleic Acids Res. 2019; 47:D853–D858. 30407534 10.1093/nar/gky1095 PMC6323953

[48] Karolchik D., Hinrichs A.S., Furey T.S., Roskin K.M., Sugnet C.W., Haussler D., Kent W.J. The UCSC Table Browser data retrieval tool. Nucleic Acids Res. 2004; 32:D493–D496. 14681465 10.1093/nar/gkh103 PMC308837

[49] Lander E.S., Linton L.M., Birren B., Nusbaum C., Zody M.C., Baldwin J., Devon K., Dewar K., Doyle M., FitzHugh W. et al. Initial sequencing and analysis of the human genome. Nature. 2001; 409:860–921. 11237011 10.1038/35057062

[50] Wu M.C., Lee S., Cai T., Li Y., Boehnke M., Lin X. Rare-variant association testing for sequencing data with the sequence kernel association test. Am. J. Hum. Genet. 2011; 89:82–93. 21737059 10.1016/j.ajhg.2011.05.029 PMC3135811

[51] Lee S., Emond M.J., Bamshad M.J., Barnes K.C., Rieder M.J., Nickerson D.A. NHLBI GO Exome Sequencing Project—ESP Lung Project Team NHLBI GO Exome Sequencing Project—ESP Lung Project Team Christiani D.C., Wurfel M.M., Lin X. Optimal unified approach for rare-variant association testing with application to small-sample case-control whole-exome sequencing studies. Am. J. Hum. Genet. 2012; 91:224–237. 22863193 10.1016/j.ajhg.2012.06.007 PMC3415556

[52] Stephens M., Balding D.J. Bayesian statistical methods for genetic association studies. Nat. Rev. Genet. 2009; 10:681–690. 19763151 10.1038/nrg2615

[53] Marchini J., Howie B., Myers S., McVean G., Donnelly P. A new multipoint method for genome-wide association studies by imputation of genotypes. Nat. Genet. 2007; 39:906–913. 17572673 10.1038/ng2088

[54] The Wellcome Trust Case Control Consortium Maller J.B., McVean G., Byrnes J., Vukcevic D., Palin K., Su Z., Howson J.M.M., Auton A., Myers S. et al. Bayesian refinement of association signals for 14 loci in 3 common diseases. Nat. Genet. 2012; 44:1294–1301. 23104008 10.1038/ng.2435 PMC3791416

[55] Kichaev G., Roytman M., Johnson R., Eskin E., Lindström S., Kraft P., Pasaniuc B. Improved methods for multi-trait fine mapping of pleiotropic risk loci. Bioinforma. Oxf. Engl. 2017; 33:248–255. 10.1093/bioinformatics/btw615 PMC5254076 27663501

[56] Hozo S.P., Djulbegovic B., Hozo I. Estimating the mean and variance from the median, range, and the size of a sample. BMC Med. Res. Methodol. 2005; 5:13. 15840177 10.1186/1471-2288-5-13 PMC1097734

[57] GTEx Consortium The Genotype-Tissue Expression (GTEx) pilot analysis: multitissue gene regulation in humans. Science. 2015; 348:648–660. 25954001 10.1126/science.1262110 PMC4547484

[58] ENCODE Consortium The ENCODE (ENCyclopedia Of DNA Elements) Project. Science. 2004; 306:636–640. 15499007 10.1126/science.1105136

[59] ENCODE Project Consortium An integrated encyclopedia of DNA elements in the human genome. Nature. 2012; 489:57–74. 22955616 10.1038/nature11247 PMC3439153

[60] Wang Y., Song F., Zhang B., Zhang L., Xu J., Kuang D., Li D., Choudhary M.N.K., Li Y., Hu M. et al. The 3D Genome Browser: a web-based browser for visualizing 3D genome organization and long-range chromatin interactions. Genome Biol. 2018; 19:151. 30286773 10.1186/s13059-018-1519-9 PMC6172833

[61] Nachman M.W., Crowell S.L. Estimate of the mutation rate per nucleotide in humans. Genetics. 2000; 156:297–304. 10978293 10.1093/genetics/156.1.297 PMC1461236

[62] Zhao Z., Boerwinkle E. Neighboring-nucleotide effects on single nucleotide polymorphisms: a study of 2.6 million polymorphisms across the human genome. Genome Res. 2002; 12:1679–1686. 12421754 10.1101/gr.287302 PMC187558

[63] Kitts A., Phan L., Ward M., Holmes J.B. The Database of Short Genetic Variation (dbSNP) National Center for Biotechnology Information (US). 2014;

[64] Niewold T.B. Advances in lupus genetics. Curr. Opin. Rheumatol. 2015; 27:440–447. 26218512 10.1097/BOR.0000000000000205 PMC4540605

[65] Patel Z.H., Lu X., Miller D., Forney C.R., Lee J., Lynch A., Schroeder C., Parks L., Magnusen A.F., Chen X. et al. A plausibly causal functional lupus-associated risk variant in the STAT1-STAT4 locus. Hum. Mol. Genet. 2018; 27:2392–2404. 29912393 10.1093/hmg/ddy140 PMC6005081

[66] Parvin J.D., Sharp P.A. DNA topology and a minimal set of basal factors for transcription by RNA polymerase II. Cell. 1993; 73:533–540. 8490964 10.1016/0092-8674(93)90140-l

[67] Scaffidi P., Bianchi M.E. Spatially precise DNA bending is an essential activity of the Sox2 transcription factor. J. Biol. Chem. 2001; 276:47296–47302. 11584012 10.1074/jbc.M107619200

[68] Kumasaka N., Knights A.J., Gaffney D.J. High-resolution genetic mapping of putative causal interactions between regions of open chromatin. Nat. Genet. 2019; 51:128–137. 30478436 10.1038/s41588-018-0278-6 PMC6330062

[69] Yang J., Fritsche L.G., Zhou X., Abecasis G. A scalable bayesian method for integrating functional information in genome-wide association studies. Am. J. Hum. Genet. 2017; 101:404–416. 28844487 10.1016/j.ajhg.2017.08.002 PMC5590971

