# Supplementary material for: Intrinsic DNA topology as a prioritization metric in genomic fine-mapping studies
Source: Nucleic Acids Res. 2020 Oct 21;48(20):11304–21. doi: 10.1093/nar/gkaa877 (PMC7672465; doi:10.1093/nar/gkaa877)

Figure S1. MPRA Log$_2$ fold change versus ∆MGW for 116 regions.

Scatterplots for 116 regions analyzed by MRPA for allelic skewing. Regions are presented and numbered by decreasing correlation estimate (r) between ∆MGW and the absolute value of Log$_2$ fold change (MPRA). The top SNP for each region refers to the SNP with the largest log$_2$ fold change (absolute value) as defined in MPRA. Regions were required to have at least five SNPs with least one SNP that met FDR-significance for allelic skewing as defined in the previously published MPRA data.

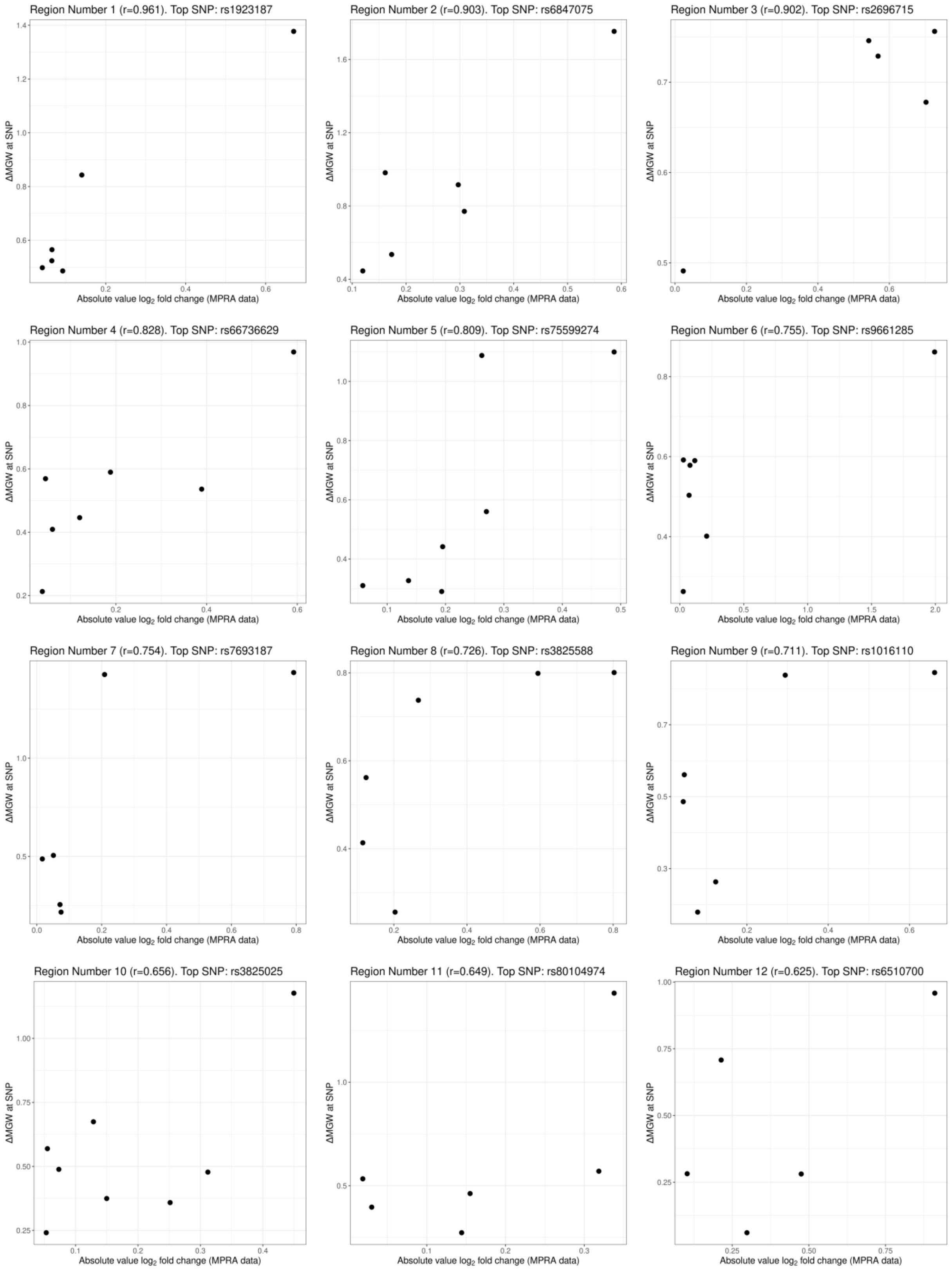

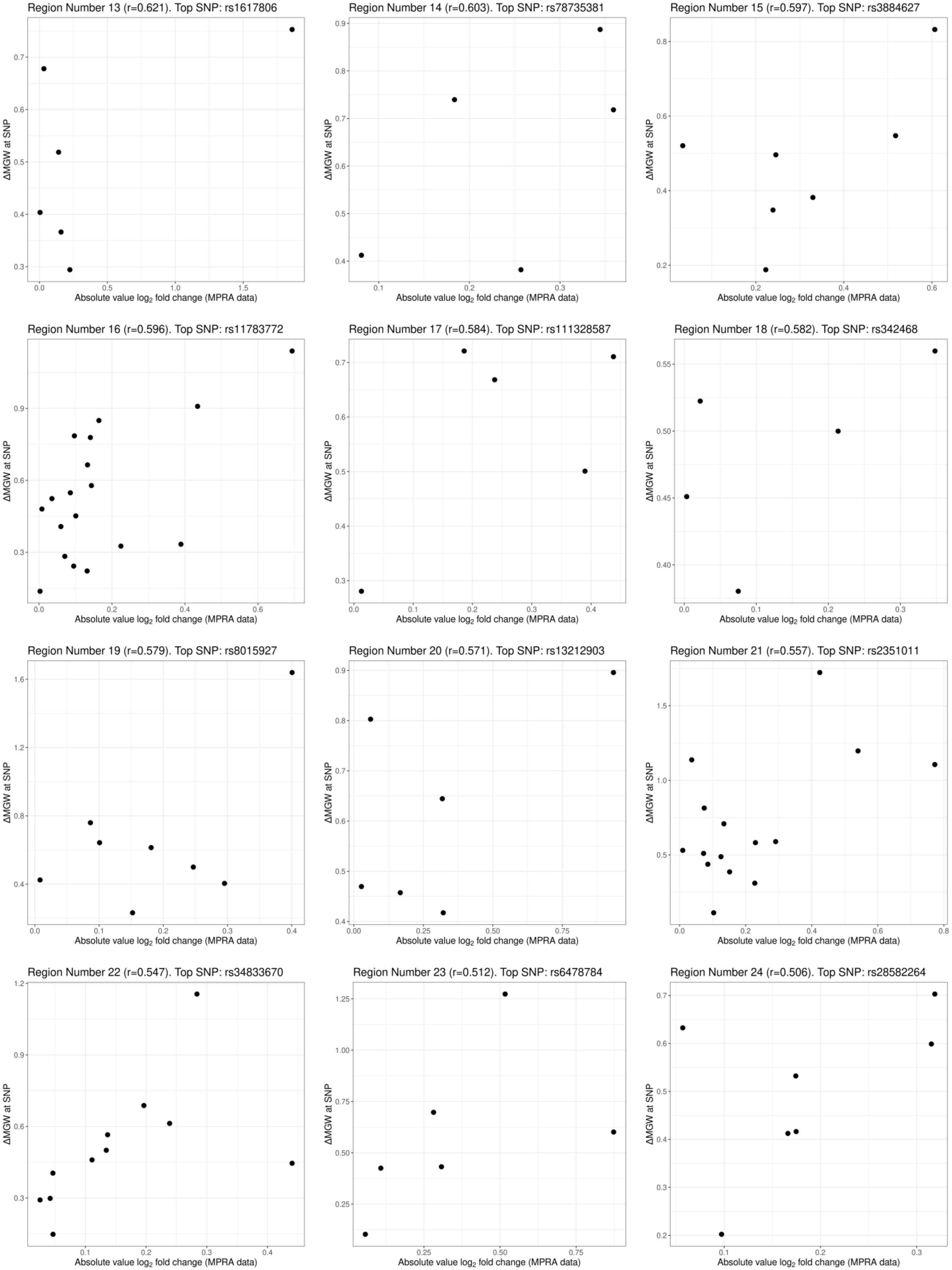
Region Number 13 (r=0.621). Top SNP: rs1617806
Region Number 14 (r=0.603). Top SNP: rs78735381
Region Number 15 (r=0.597). Top SNP: rs3884627
Region Number 16 (r=0.596). Top SNP: rs11783772
Region Number 17 (r=0.584). Top SNP: rs111328587
Region Number 18 (r=0.582). Top SNP: rs342468
Region Number 19 (r=0.579). Top SNP: rs8015927
Region Number 20 (r=0.571). Top SNP: rs13212903
Region Number 21 (r=0.557). Top SNP: rs2351011
Region Number 22 (r=0.547). Top SNP: rs34833670
Region Number 23 (r=0.512). Top SNP: rs6478784
Region Number 24 (r=0.506). Top SNP: rs28582264
ΔMGW at SNP
Absolute value log_2 fold change (MPRA data)

Region Number 25 (r=0.495). Top SNP: rs7905607

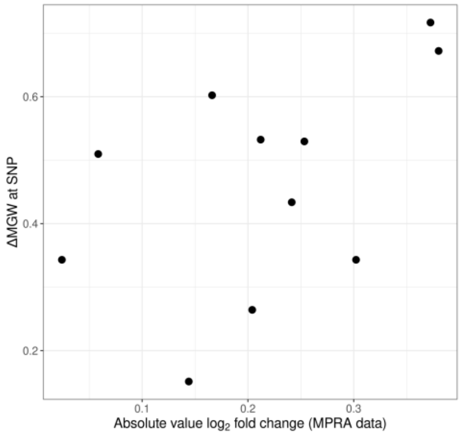

Region Number 26 (r=0.467). Top SNP: rs113859809

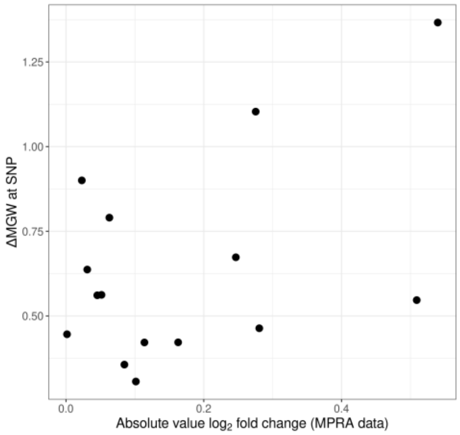

Region Number 27 (r=0.457). Top SNP: rs78720068

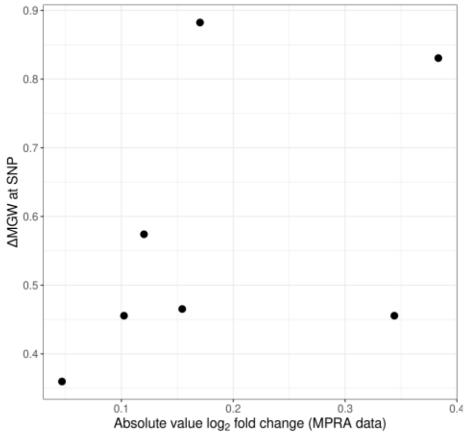

Region Number 28 (r=0.454). Top SNP: rs61435451

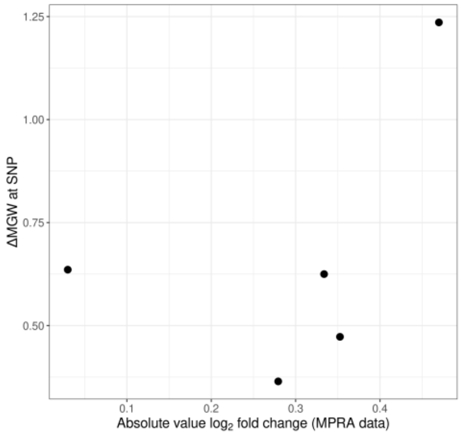

Region Number 29 (r=0.424). Top SNP: rs35883594

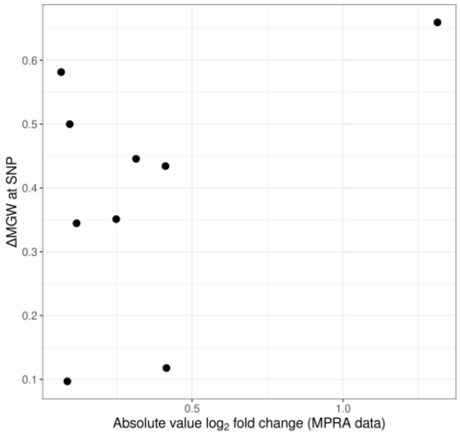

Region Number 30 (r=0.416). Top SNP: rs5016900

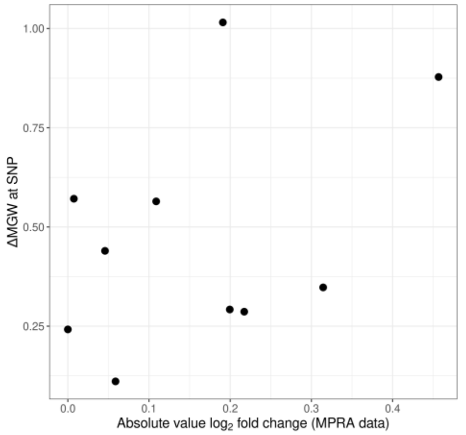

Region Number 31 (r=0.41). Top SNP: rs227578

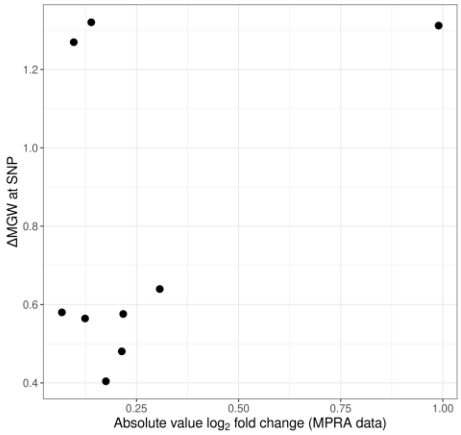

Region Number 32 (r=0.366). Top SNP: rs2858490

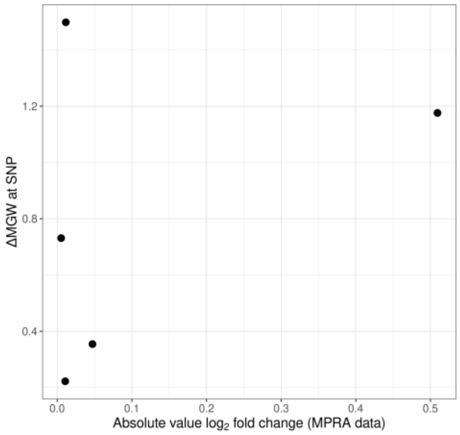

Region Number 33 (r=0.316). Top SNP: rs9450311

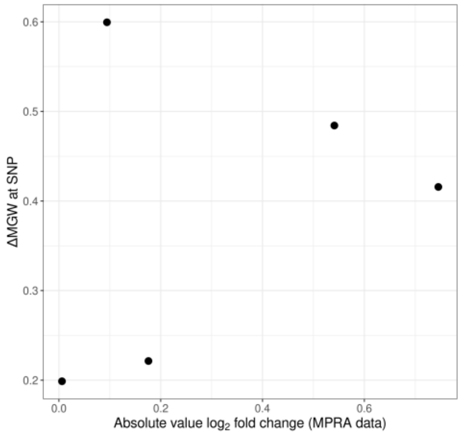

Region Number 34 (r=0.307). Top SNP: rs2015767

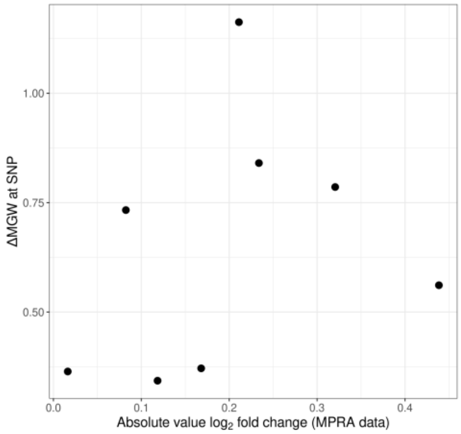

Region Number 35 (r=0.284). Top SNP: rs112561341

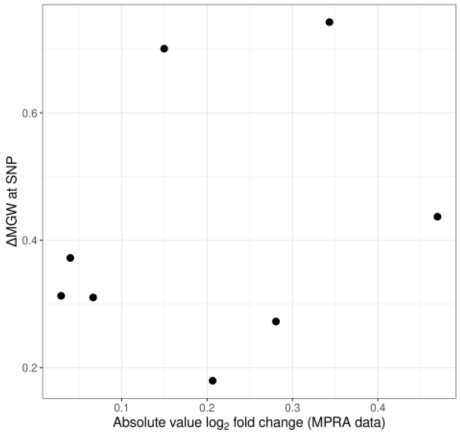

Region Number 36 (r=0.272). Top SNP: rs3796812

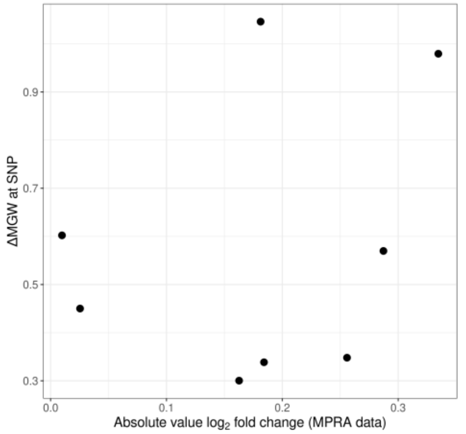

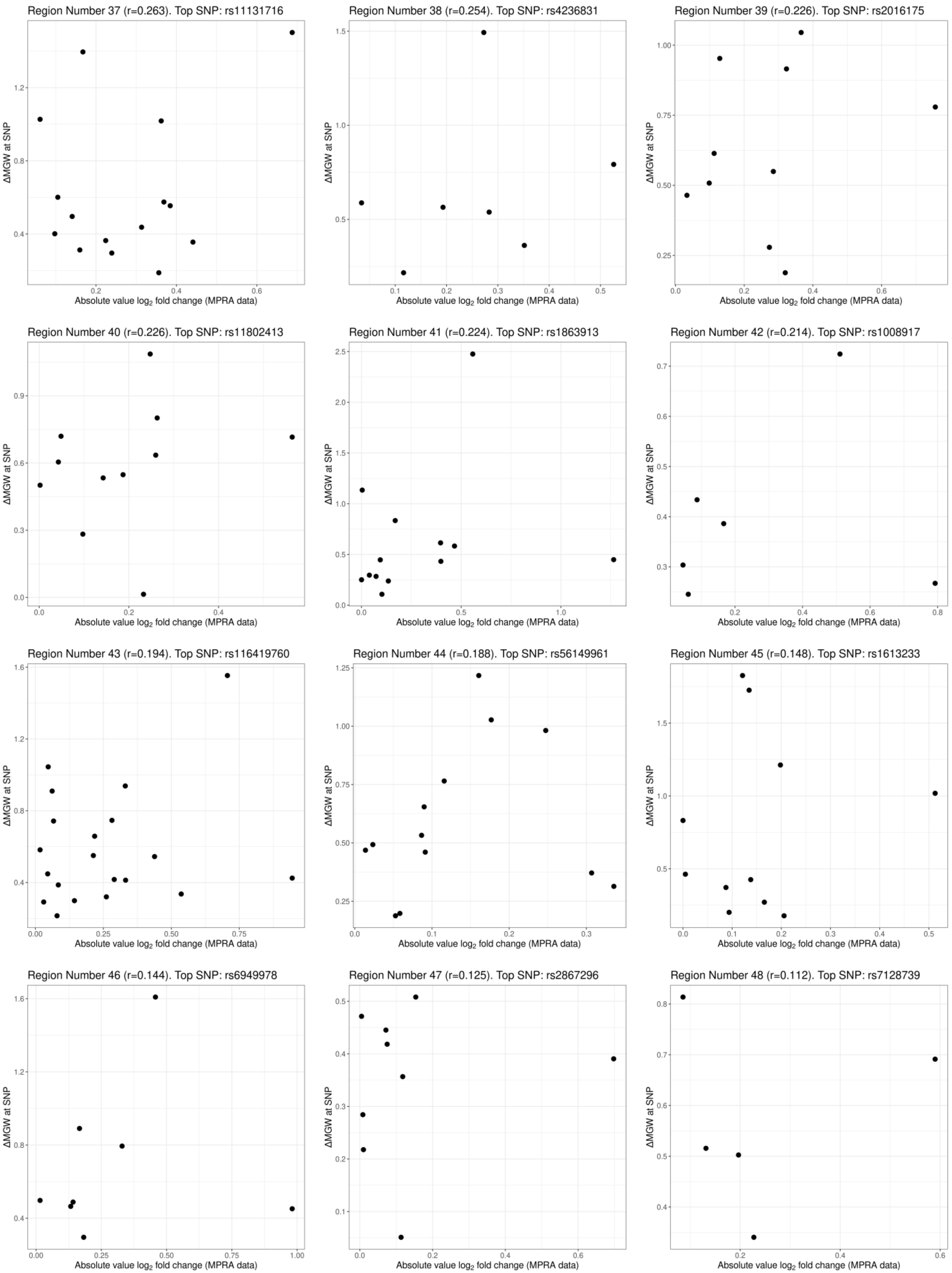
Region Number 37 (r=0.263). Top SNP: rs11131716
Region Number 38 (r=0.254). Top SNP: rs4236831
Region Number 39 (r=0.226). Top SNP: rs2016175
Region Number 40 (r=0.226). Top SNP: rs11802413
Region Number 41 (r=0.224). Top SNP: rs1863913
Region Number 42 (r=0.214). Top SNP: rs1008917
Region Number 43 (r=0.194). Top SNP: rs116419760
Region Number 44 (r=0.188). Top SNP: rs56149961
Region Number 45 (r=0.148). Top SNP: rs1613233
Region Number 46 (r=0.144). Top SNP: rs6949978
Region Number 47 (r=0.125). Top SNP: rs2867296
Region Number 48 (r=0.112). Top SNP: rs7128739
ΔMGW at SNP
Absolute value $\log_2$ fold change (MPRA data)

Region Number 49 (r=0.104). Top SNP: rs11880872

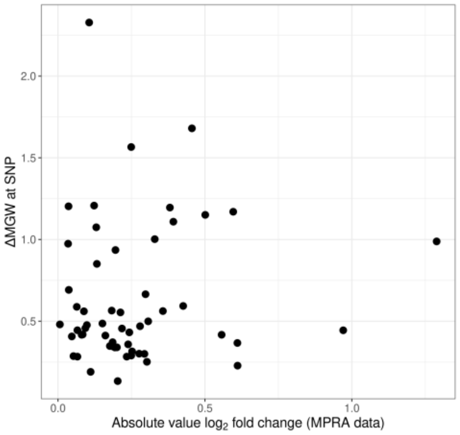

Region Number 50 (r=0.079). Top SNP: rs9393894

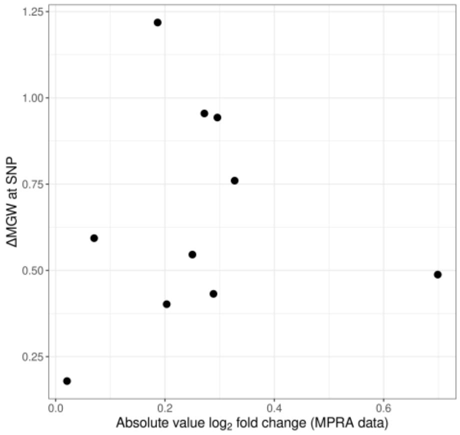

Region Number 51 (r=0.061). Top SNP: rs4793995

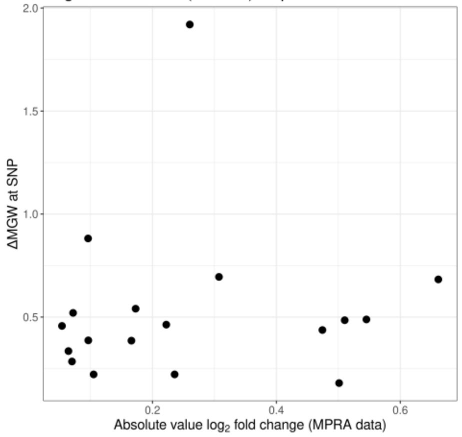

Region Number 52 (r=0.049). Top SNP: rs58726213

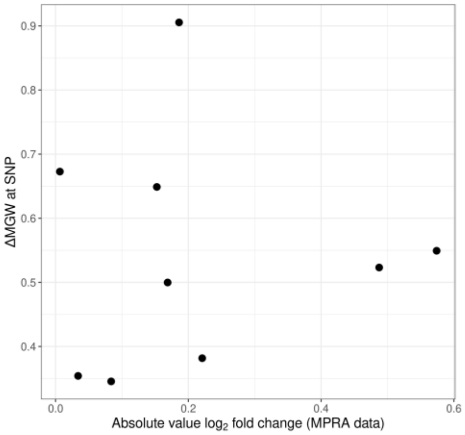

Region Number 53 (r=0.041). Top SNP: rs16949275

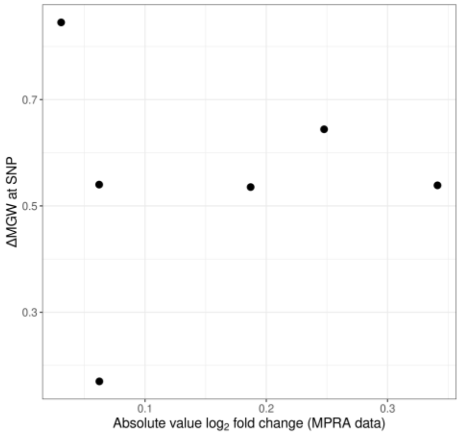

Region Number 54 (r=0.041). Top SNP: rs2258769

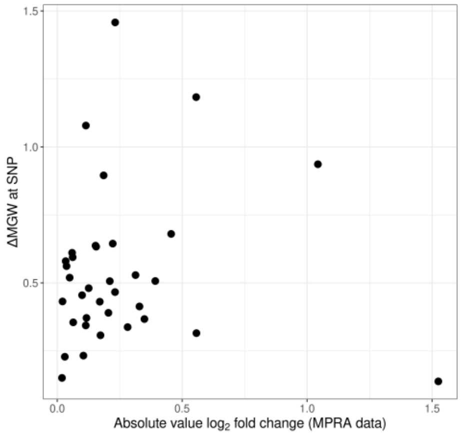

Region Number 55 (r=0.04). Top SNP: rs2878525

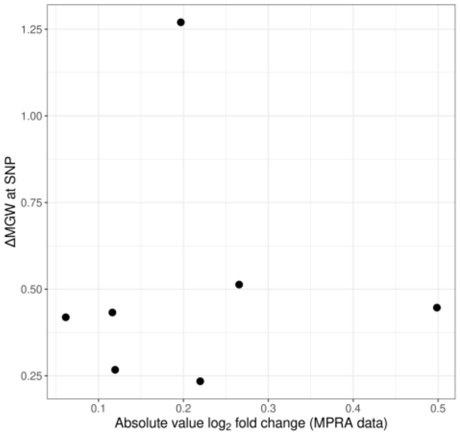

Region Number 56 (r=0.038). Top SNP: rs78434425

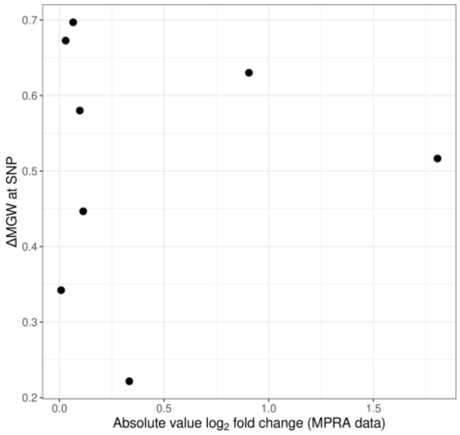

Region Number 57 (r=0.009). Top SNP: rs112636307

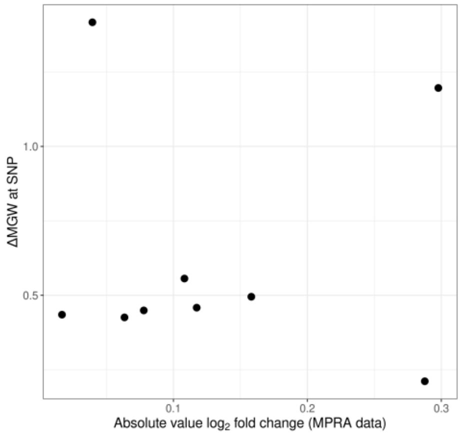

Region Number 58 (r=0.008). Top SNP: rs146833335

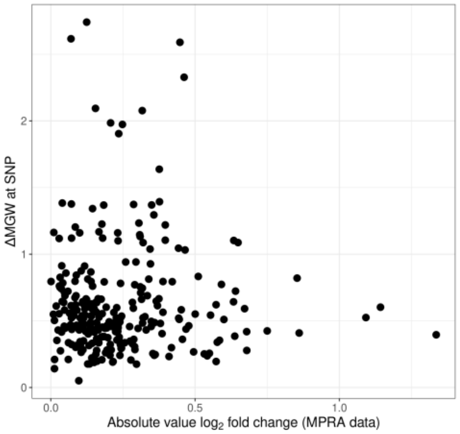

Region Number 59 (r=0.003). Top SNP: rs17185052

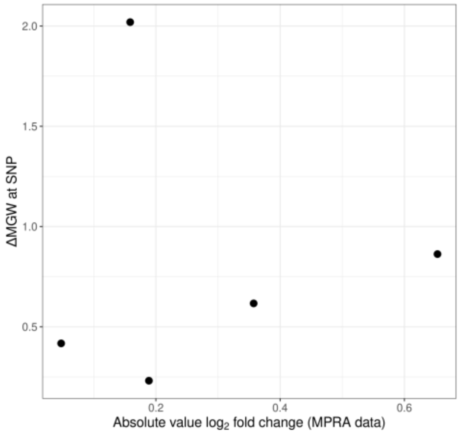

Region Number 60 (r=-0.009). Top SNP: rs62309221

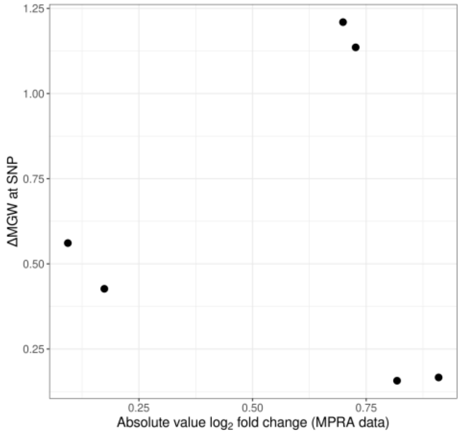

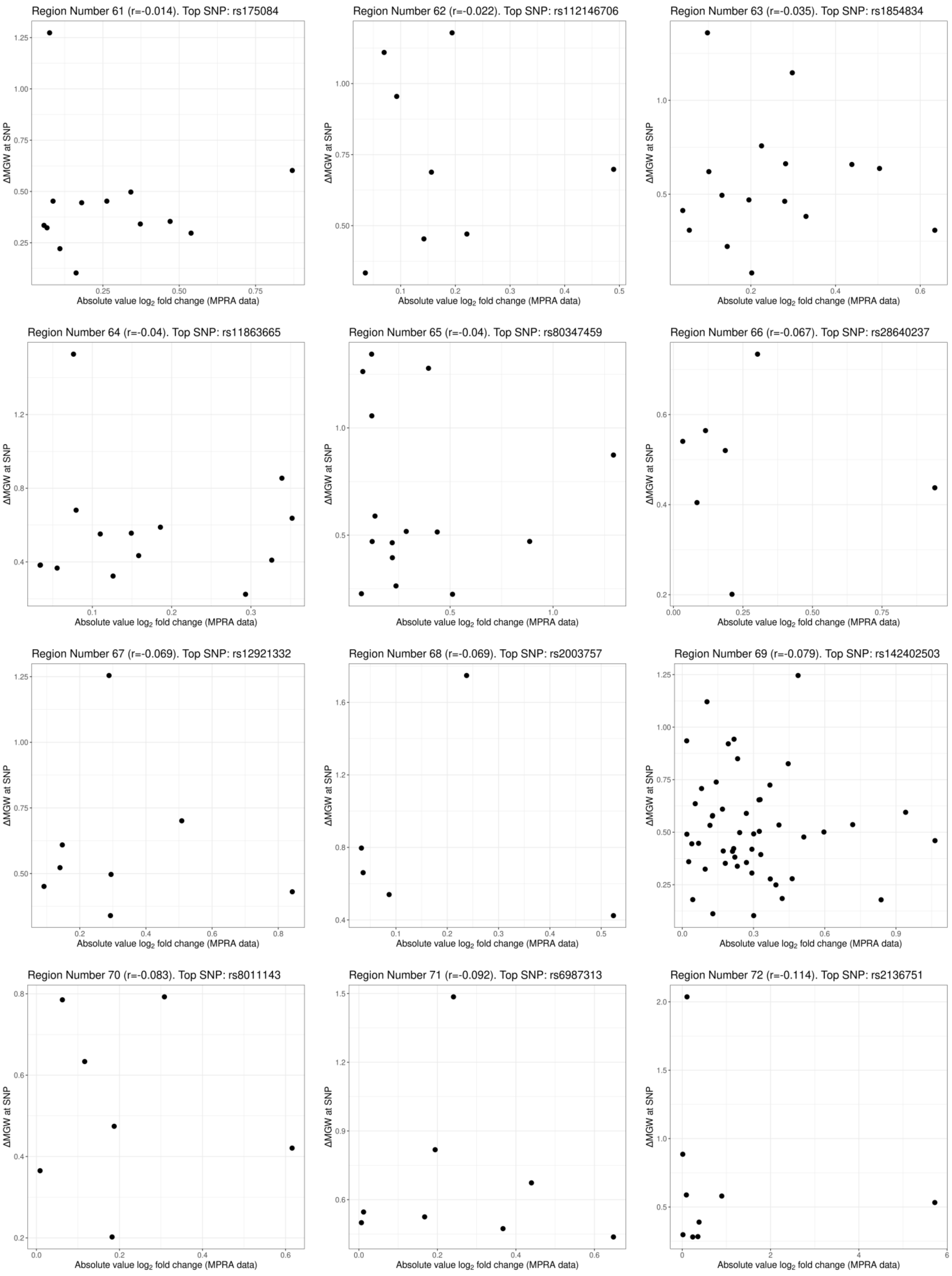

Region Number 61 (r=-0.014). Top SNP: rs175084
Region Number 62 (r=-0.022). Top SNP: rs112146706
Region Number 63 (r=-0.035). Top SNP: rs1854834
Region Number 64 (r=-0.04). Top SNP: rs11863665
Region Number 65 (r=-0.04). Top SNP: rs80347459
Region Number 66 (r=-0.067). Top SNP: rs28640237
Region Number 67 (r=-0.069). Top SNP: rs12921332
Region Number 68 (r=-0.069). Top SNP: rs2003757
Region Number 69 (r=-0.079). Top SNP: rs142402503
Region Number 70 (r=-0.083). Top SNP: rs8011143
Region Number 71 (r=-0.092). Top SNP: rs6987313
Region Number 72 (r=-0.114). Top SNP: rs2136751
ΔMGW at SNP
Absolute value $\log_2$ fold change (MPRA data)

Region Number 73 (r=-0.12). Top SNP: rs2473294

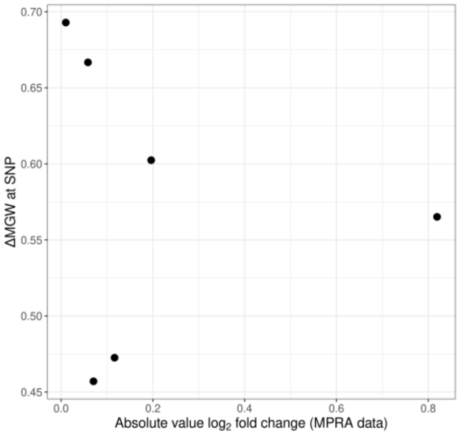

Region Number 74 (r=-0.123). Top SNP: rs28610213

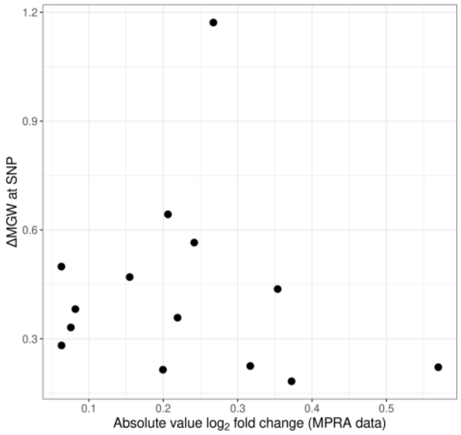

Region Number 75 (r=-0.124). Top SNP: rs62116613

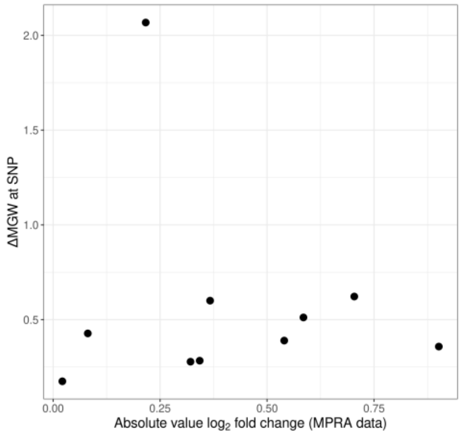

Region Number 76 (r=-0.148). Top SNP: rs115546344

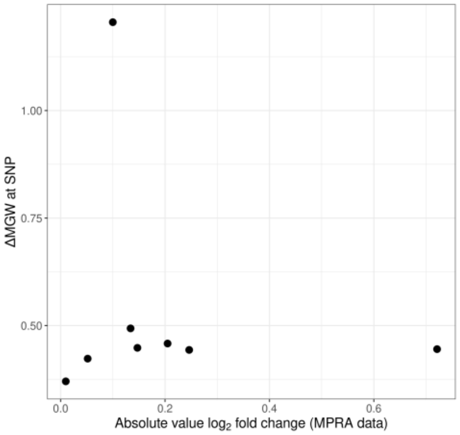

Region Number 77 (r=-0.151). Top SNP: rs12769981

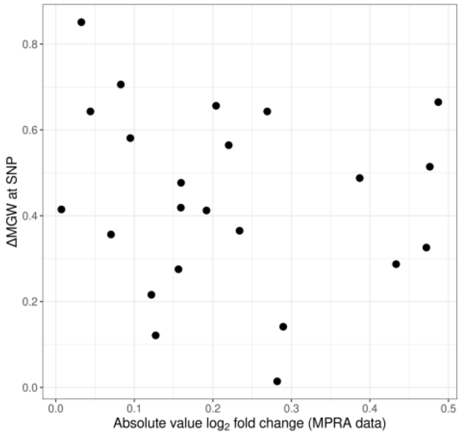

Region Number 78 (r=-0.17). Top SNP: rs11600860

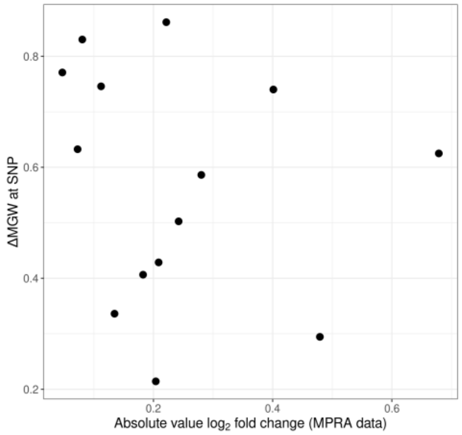

Region Number 79 (r=-0.17). Top SNP: rs3756578

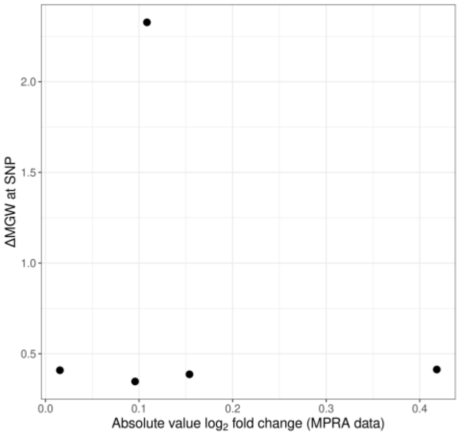

Region Number 80 (r=-0.174). Top SNP: rs6825544

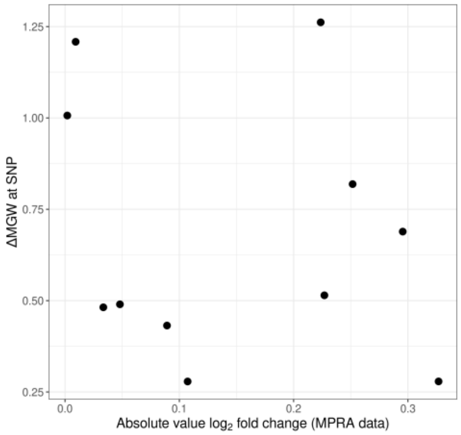

Region Number 81 (r=-0.189). Top SNP: rs183369193

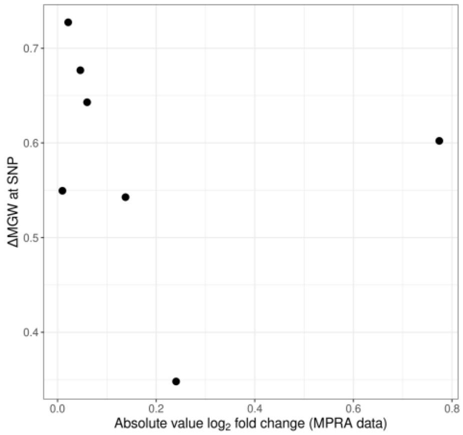

Region Number 82 (r=-0.211). Top SNP: rs555001

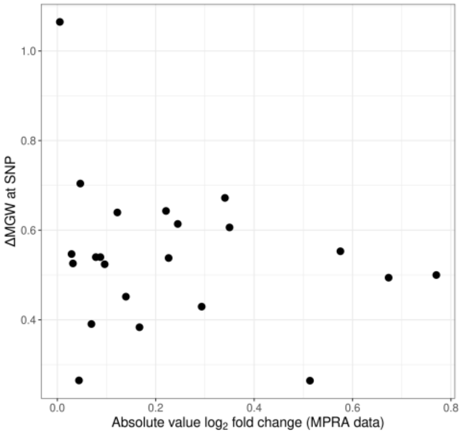

Region Number 83 (r=-0.222). Top SNP: rs4930698

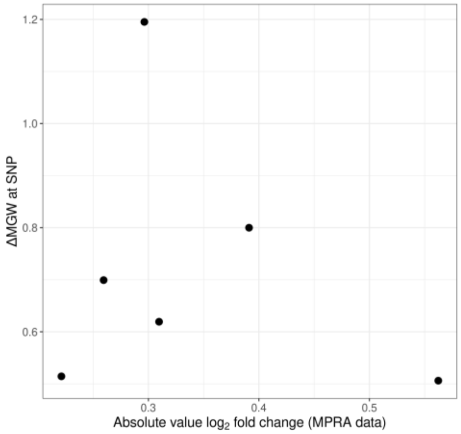

Region Number 84 (r=-0.23). Top SNP: rs77330556

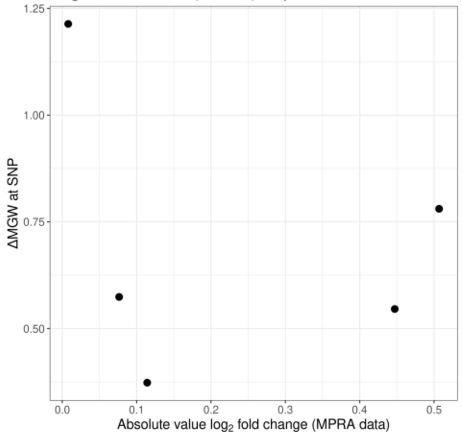

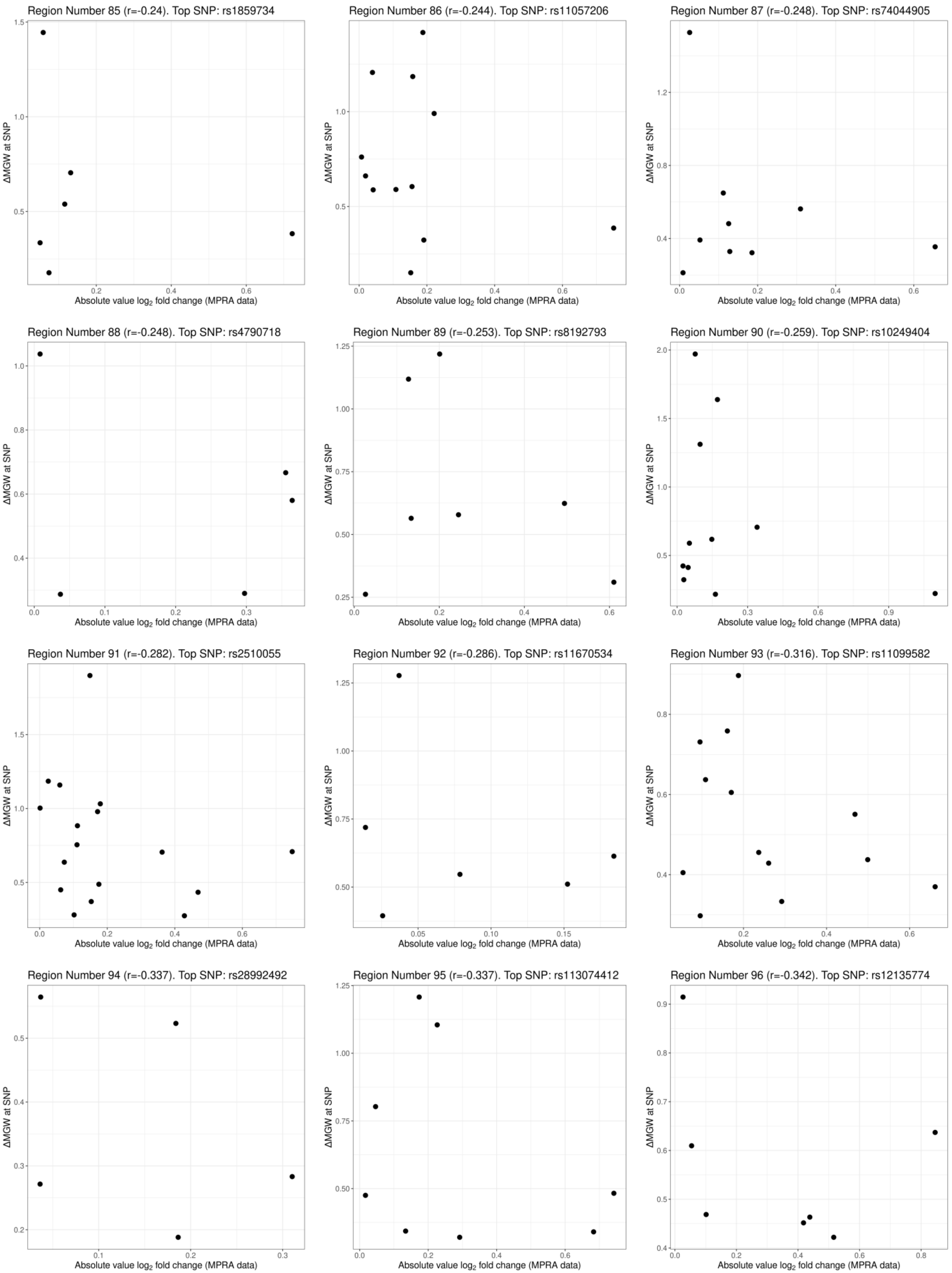

Region Number 85 (r=-0.24). Top SNP: rs1859734
Region Number 86 (r=-0.244). Top SNP: rs11057206
Region Number 87 (r=-0.248). Top SNP: rs74044905
Region Number 88 (r=-0.248). Top SNP: rs4790718
Region Number 89 (r=-0.253). Top SNP: rs8192793
Region Number 90 (r=-0.259). Top SNP: rs10249404
Region Number 91 (r=-0.282). Top SNP: rs2510055
Region Number 92 (r=-0.286). Top SNP: rs11670534
Region Number 93 (r=-0.316). Top SNP: rs11099582
Region Number 94 (r=-0.337). Top SNP: rs28992492
Region Number 95 (r=-0.337). Top SNP: rs113074412
Region Number 96 (r=-0.342). Top SNP: rs12135774
ΔMGW at SNP
Absolute value log_2 fold change (MPRA data)

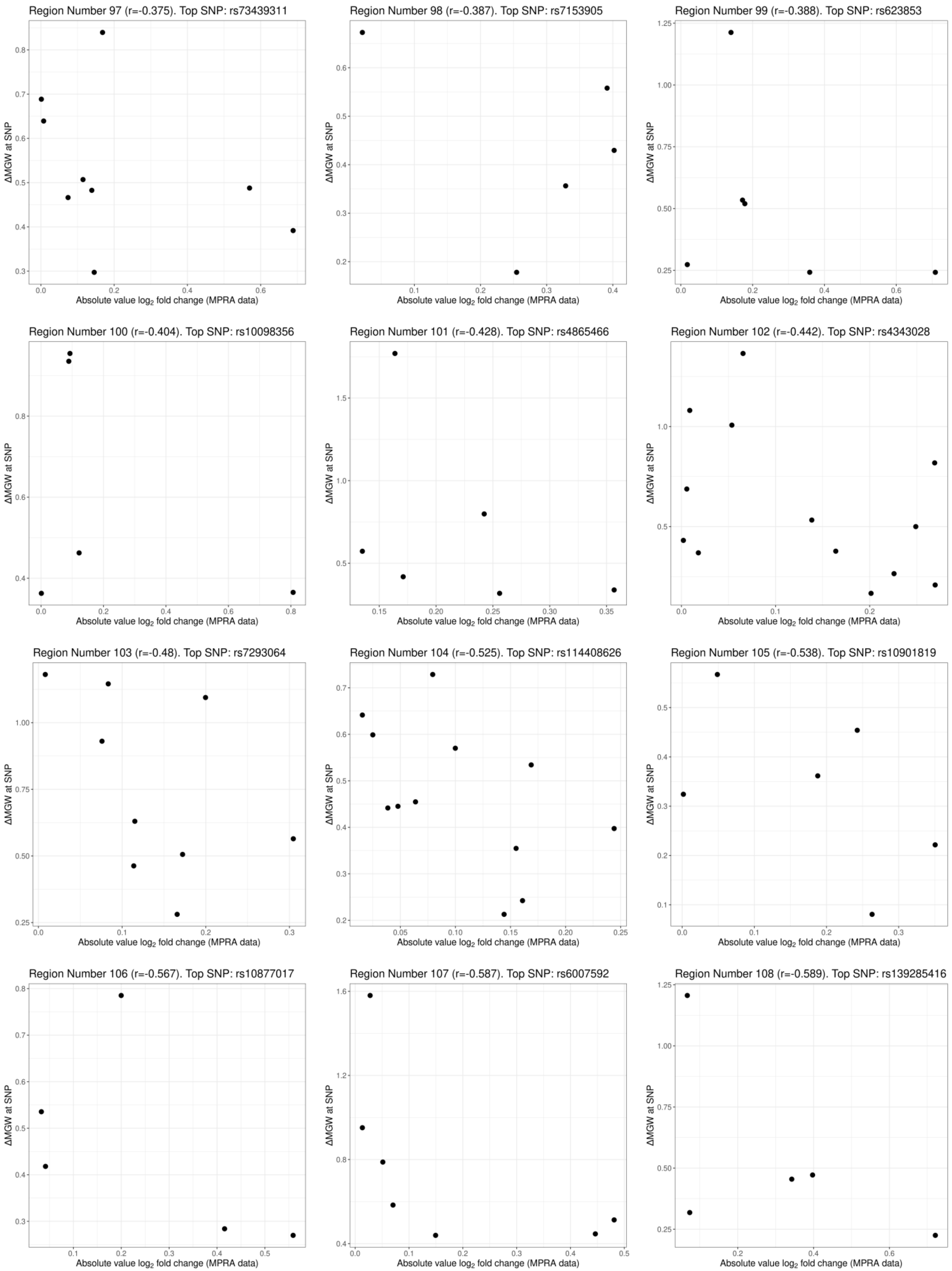
Region Number 97 (r=-0.375). Top SNP: rs73439311
Region Number 98 (r=-0.387). Top SNP: rs7153905
Region Number 99 (r=-0.388). Top SNP: rs623853
Region Number 100 (r=-0.404). Top SNP: rs10098356
Region Number 101 (r=-0.428). Top SNP: rs4865466
Region Number 102 (r=-0.442). Top SNP: rs4343028
Region Number 103 (r=-0.48). Top SNP: rs7293064
Region Number 104 (r=-0.525). Top SNP: rs114408626
Region Number 105 (r=-0.538). Top SNP: rs10901819
Region Number 106 (r=-0.567). Top SNP: rs10877017
Region Number 107 (r=-0.587). Top SNP: rs6007592
Region Number 108 (r=-0.589). Top SNP: rs139285416
ΔMGW at SNP
Absolute value log_2 fold change (MPRA data)

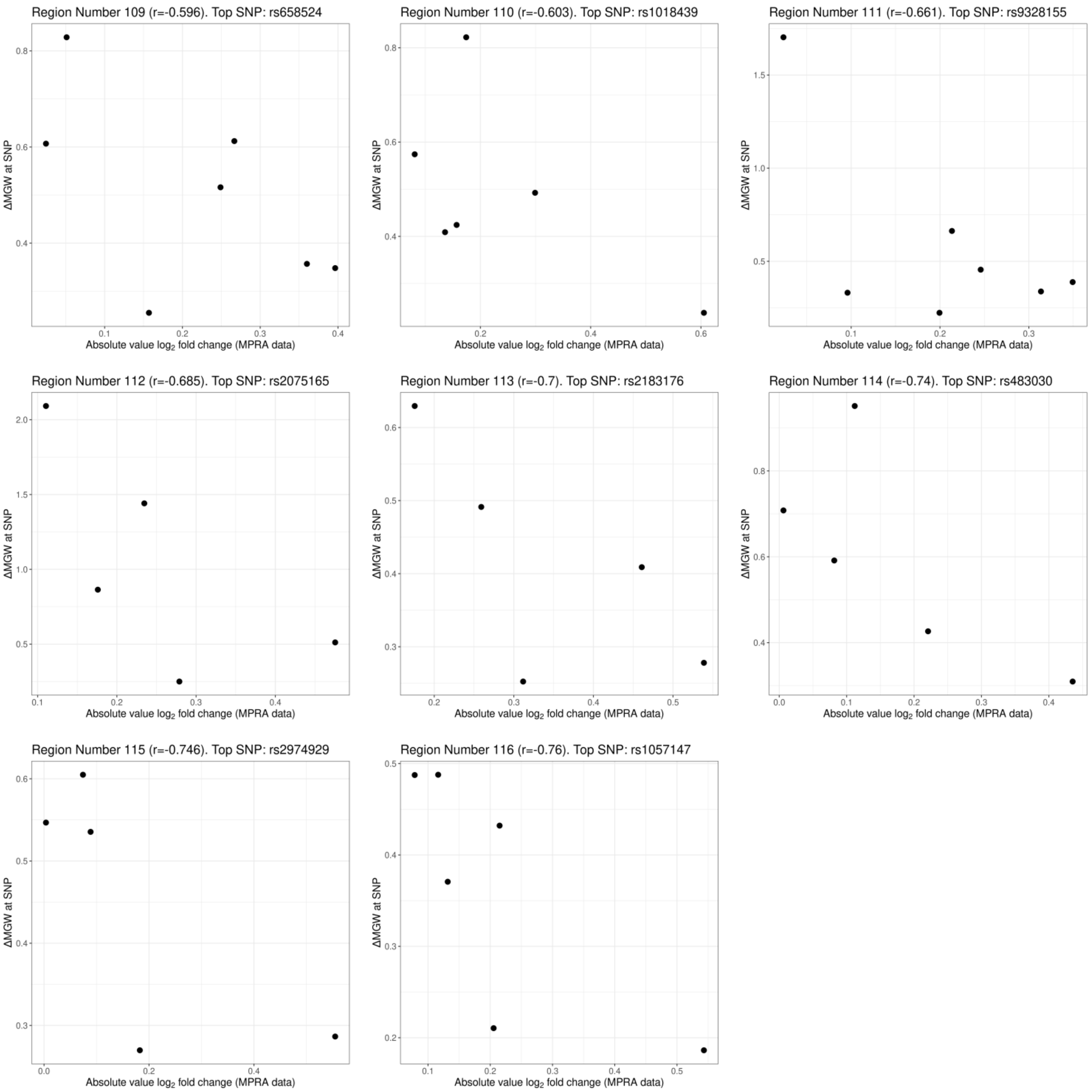
Region Number 109 (r=-0.596). Top SNP: rs658524
Region Number 110 (r=-0.603). Top SNP: rs1018439
Region Number 111 (r=-0.661). Top SNP: rs9328155
Region Number 112 (r=-0.685). Top SNP: rs2075165
Region Number 113 (r=-0.7). Top SNP: rs2183176
Region Number 114 (r=-0.74). Top SNP: rs483030
Region Number 115 (r=-0.746). Top SNP: rs2974929
Region Number 116 (r=-0.76). Top SNP: rs1057147
ΔMGW at SNP
Absolute value log2 fold change (MPRA data)

Figure S2. SKAT and SKAT-O analyses in *FAM167A-BLK*.

Comparison of results for the *FAM167A-BLK* region using SKAT in a ΔMGW-weighted and equally weighted analysis. SNPs prioritized in the unweighted analyses (blue diamonds) were those that had multiple SNPs from the highly-associated LD block (Figure 6) within the same 5-SNP analysis windows (Supplementary Table S5-S6). Weighted analysis (shown in purple) instead prioritized SNPs that had larger ΔMGW and association values. This shifted the signal upstream to rs2061831. We note that the SKAT-O analysis performed very similarly to SKAT.

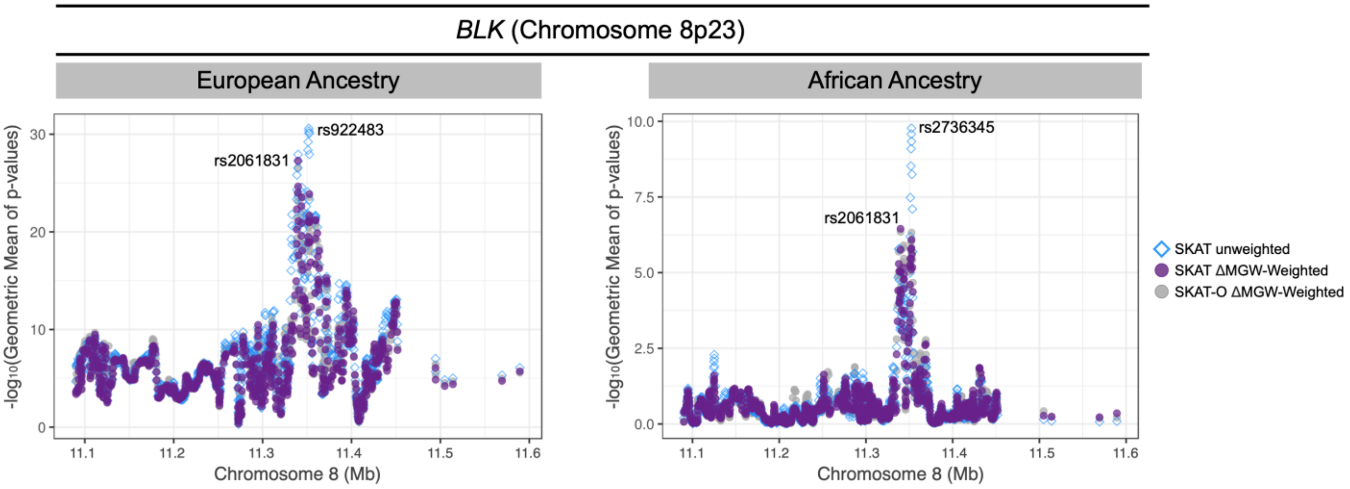

Figure S3. ENCODE SCREEN information for cCREs at SNPs identified in *FAM167A-BLK* analyses.

SNPs rs2061831, rs13277113, and rs2736440 were searched in the GRch38 ENCODE SCREEN database (screen.encodeproject.org) to identify if any SNPs resided within credible Cis-regulatory elements (cCREs). Only rs2061831 and rs13277113 yielded cCREs. Screen captures of the data are shown.

A SCREEN GRch38 results

Queried SNP	ENCODE SCREEN cCRE accession	DNase max-Z	H3K4me3 max-Z	H3K27ac max-Z	CTCF max-Z	cCRE coordinates(GRch38) chr	start	length
rs2061831	EH38E2610769 D	3.84	1.57	3.83	3.79	chr8	11,482,124	288
rs13277113	EH38E2610775 D	2.82	2.35	4.53	1.63	chr8	11,491,413	311

High H3K4me3
High H3K27ac
High CTCF
High DNase
Z-score < 1.64
No data
P/D Proximal/Distal to a Transcription Start Site

B TFs that bind this cCRE

EH38E2610769

factor	# of experiments that support TF binding
CTCF	11
RAD21	2
EED	1
YY1	1
TAF1	1
RUNX3	1
POLR2A	1
ZNF687	1
BHLHE40	1

EH38E2610775

factor	# of experiments that support TF binding
YY1	1
HDAC2	1
ZNF217	1
SMARCA5	1

C Histone Marks at this cCRE

EH38E2610769

mark	# of experiments that support histone modification
H3K4me1	38
H3K27ac	20
H3K27me3	12
H3K4me2	6
H2AFZ	4
H3K9ac	2
H4K8ac	2
H3K4ac	1
H4K5ac	1
H2AK5ac	1

EH38E2610775

mark	# of experiments that support histone modification
H3K4me1	20
H3K27ac	18
H3K4me2	9
H3K4me3	9
H3K27me3	7
H3K9ac	5
H3F3A	4
H3K9me3	4
H2AFZ	3

Figure S4. Chromatin interactions (Hi-C) across cell types for identified SNPs in *FAM167A-BLK*.

Comparison of Hi-C interactions for three SNPs in the *BLK-FAM167A* region. Data was queried using the 3D genome browser (http://promoter.bx.psu.edu/hi-c/). Each plot is centered on the labeled SNP with +/-500 kb. Each arc indicates a chromatin interaction, as observed from Hi-C data. Chromatin interactions are only shown for those that have either a start or stop region that overlaps with the labeled SNP. The number of chromatin interactions with a SNP's region are indicated in parentheses for each plot. A majority of the observed chromatin interactions occur within 500 kb, with only a few extending further than the plotted region (e.g. rs2061831 for B-Cells, CD4-Cells, CD8-Cells, and Monocytes. For rs2736340, no chromatin interactions were observed in CD4-cells, CD8-cells, and Neutrophils. rs2061831 was strongly prioritized across ΔMGW-weighted analyses in both European and African Ancestries; and this SNP shows the most interactions for this data. The other two SNPs were identified in single-ancestry association analyses. Rs13277113 was the top SNP in EA while rs2736340 was the top SNP in AA analyses. Although all three SNPs are in high LD, they are physically separated from one another, eliciting different patterns of chromatin interactions.

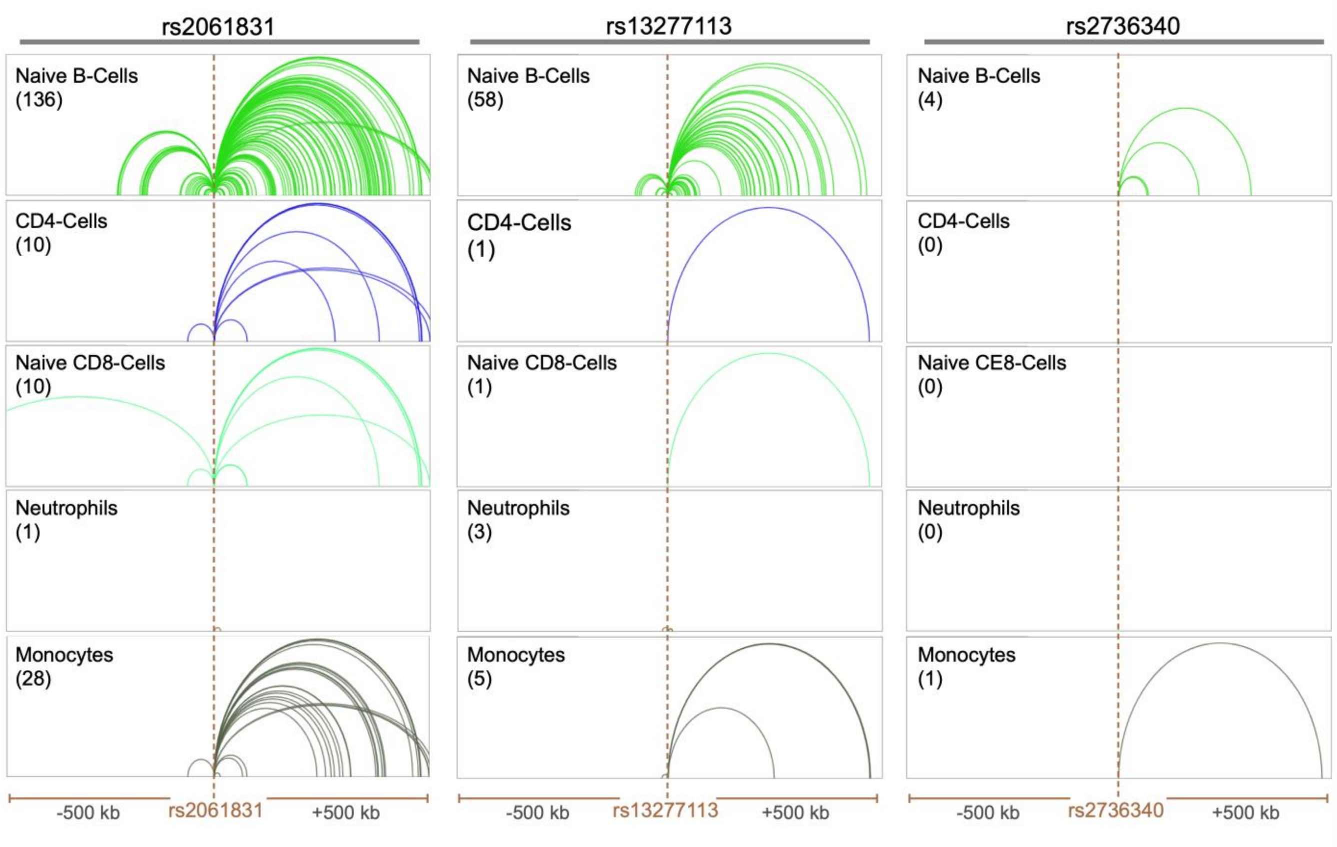

Figure S5. SKAT and SKAT-O analyses in *STAT4*.

Comparison of results for the *STAT4* region using SKAT in a ΔMGW-weighted (purple dots) and equally weighted analysis (blue diamonds). We note that the SKAT-O analysis performed very similarly to SKAT

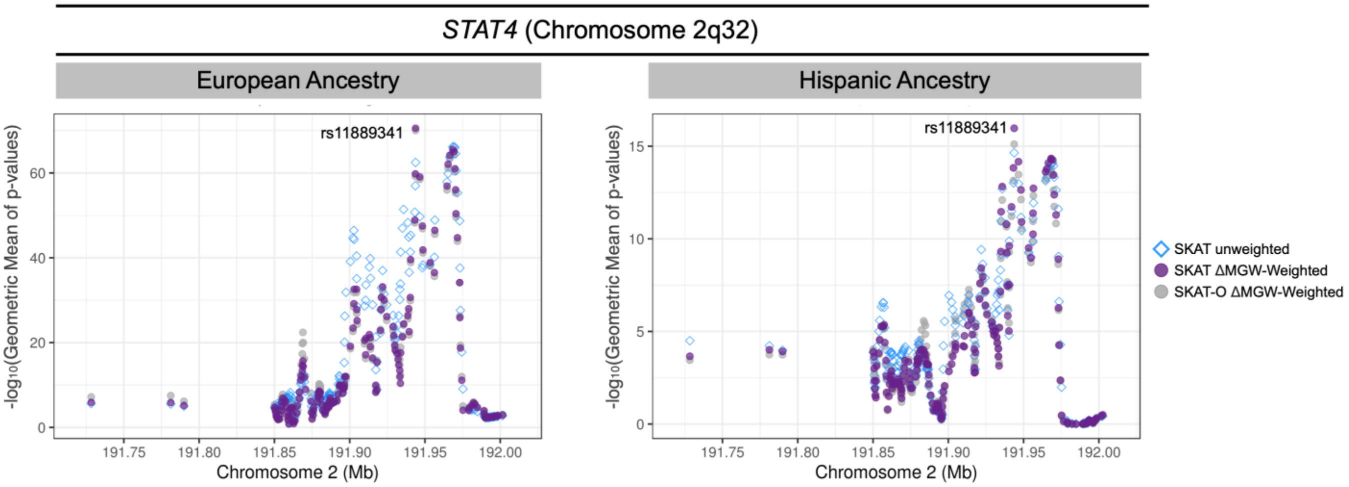

Figure S6. SKAT and SKAT-O analyses in *TNIP1*.

Comparison of results for the *TNIP1* region using SKAT in a ΔMGW-weighted and equally weighted analysis. Here, ΔMGW did not distinguish SNPs differently from the unweighted-analysis, other than an overall diminished prioritization signal, which is consistent for the low magnitudes of ΔMGW observed for the top-associated SNPs in the single-logistic regression analyses (Supplementary Table S11-S12). In this region, SLE association, not ΔMGW, was the driver for prioritizing SNPs. We note that the SKAT-O analysis performed very similarly to SKAT

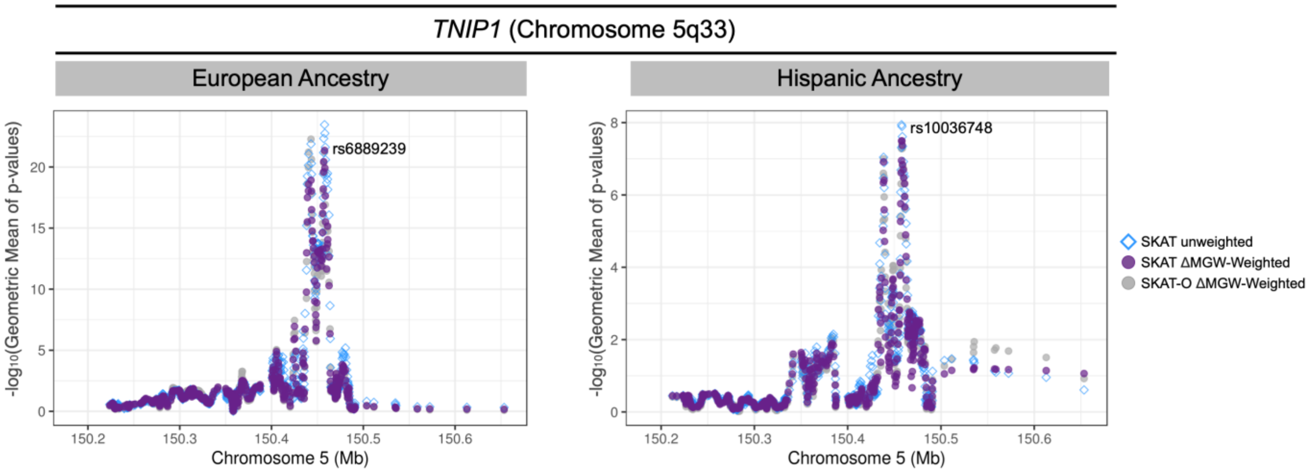

Supplement: gkaa877_Supplemental_Files [file gkaa877_supplemental_files.zip › 02_supplementary_figures_Oct5th.pdf]
